# Supplementary material for: Metabolic control of CD47 expression through LAT2-mediated amino acid uptake promotes tumor immune evasion
Source: Nat Commun. 2022 Oct 23;13:6308. doi: 10.1038/s41467-022-34064-4 (PMC9588779; doi:10.1038/s41467-022-34064-4)
Supplement: Supplementary file 1 — Supplementary Information [file 41467_2022_34064_MOESM1_ESM.pdf]

# Supplementary Information

## Metabolic control of CD47 expression through LAT2-mediated amino acid uptake promotes tumor immune evasion

Zenan Wang[1,2,3#], Binghao Li[1,2,3#], Shan Li[4#], Wenlong Lin[1,5], Zhan Wang[1,2,3], Shengdong Wang[1,2,3], Weida Chen[1], Wei Shi[1], Tao Chen[1,2,3], Hao Zhou[1,2,3], Eloy Yinwang[1,2,3], Wenkan Zhang[1,2,3], Haochen Mou[1,2,3], Xupeng Chai[1,2,3], Jiahao Zhang[1,2,3], Zhimin Lu[4,6], Zhaoming Ye[1,2,3*]

1.Department of Orthopedic Surgery, The Second Affiliated Hospital, Zhejiang University School of Medicine, Hangzhou, Zhejiang, China
2. Orthopedics Research Institute of Zhejiang University, Hangzhou, Zhejiang, China
3. Key Laboratory of Motor System Disease Research and Precision Therapy of Zhejiang Province, Hangzhou, Zhejiang, China
4. Department of Hepatobiliary and Pancreatic Surgery and Zhejiang Provincial Key Laboratory of Pancreatic Disease of The First Affiliated Hospital, Institute of Translational Medicine, Zhejiang University School of Medicine, Hangzhou, Zhejiang, China
5. Institute of Immunology, Zhejiang University School of Medicine, Hangzhou, Zhejiang, China
6. Zhejiang University Cancer Center, Hangzhou, Zhejiang, China

#Zenan Wang, Binghao Li and Shan Li contributed equally to this work.

*Corresponding author: Zhaoming Ye

## Supplementary figures and figure legends

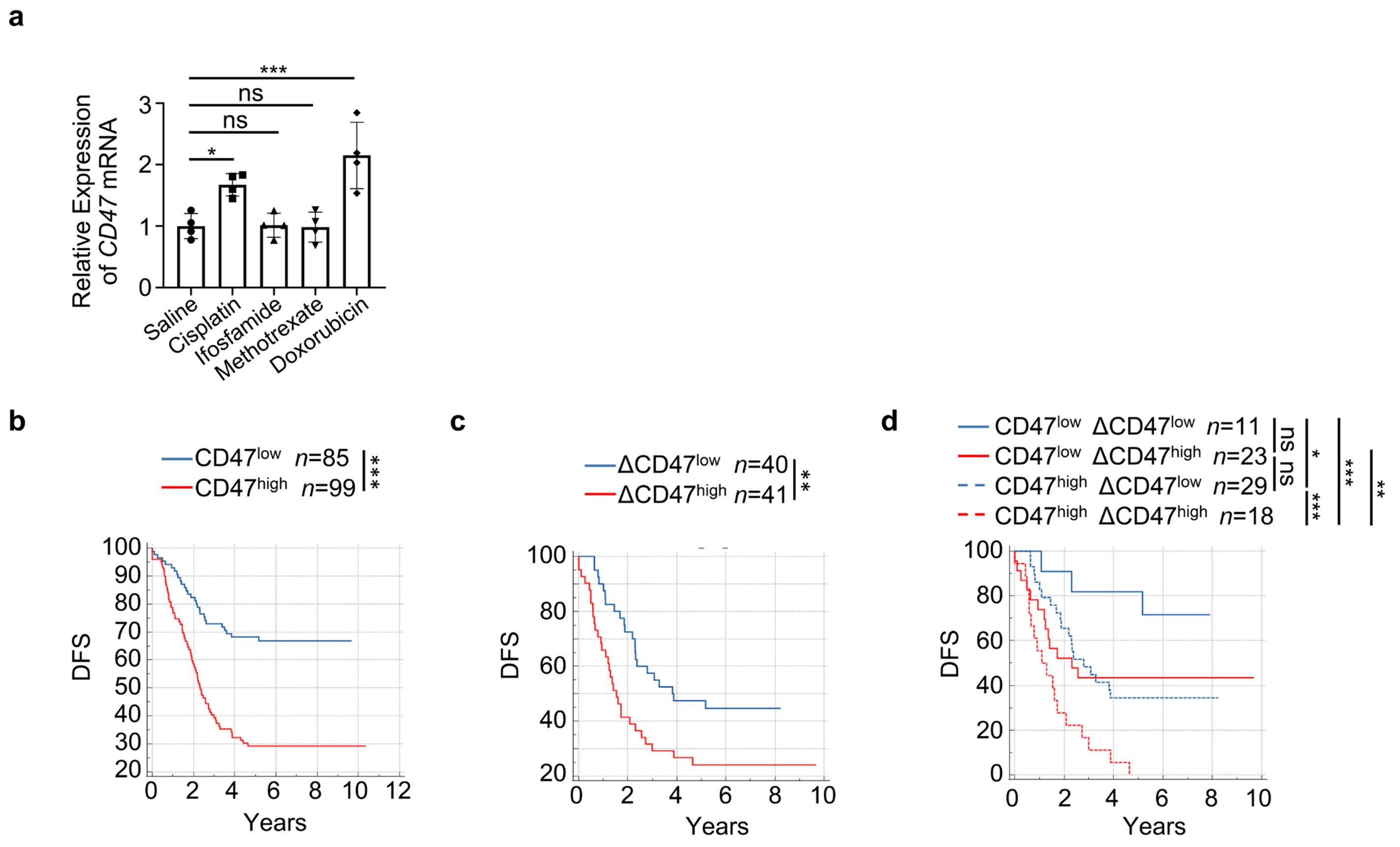


**Supplementary Figure 1, related to Fig. 1. Osteosarcoma chemotherapy induces CD47 upregulation, which is associated with poor prognosis for patients.**

**a** Quantitative real-time PCR analysis of CD47 expression in HOS tumors treated as described in **Fig. 1b, c** (*n*=4 mice per group).

**b** Kaplan–Meier curves of disease-free survival (DFS) of osteosarcoma patients stratified by CD47 expression in pre-chemotherapy specimens. Comparison was made between groups with high CD47 expression (CD47$^{high}$; CD47 H-score > 0.9) and low CD47 expression (CD47$^{low}$; CD47 H-score ≤ 0.9).

**c** Kaplan–Meier curves of disease-free survival (DFS) of osteosarcoma patients stratified by ΔCD47 expression. Comparison was made between groups with high ΔCD47 expression (ΔCD47$^{high}$; ΔCD47 H-score > 0.7) and low ΔCD47 expression (ΔCD47$^{low}$; ΔCD47 H-score < 0.7). ΔCD47 H-scores for each patient was obtained from subtracting CD47 H-score of pre-chemotherapy specimens from that of paired post-chemotherapy specimens.

**d** Kaplan–Meier curves of disease-free survival (DFS) of osteosarcoma patients stratified by CD47 expression in pre-chemotherapy specimens and ΔCD47 expression post-chemotherapy. Comparison was made among groups with low CD47 expression and low ΔCD47 expression (CD47 H-score ≤ 0.9 and ΔCD47 H-score < 0.7), low CD47 expression and high ΔCD47 expression (CD47 H-score ≤ 0.9 and ΔCD47 H-score > 0.7), high CD47 expression and low ΔCD47 expression (CD47 H-score > 0.9 and ΔCD47 H-score < 0.7), and high CD47 expression and high ΔCD47 expression (CD47 H-score > 0.9 and ΔCD47 H-score > 0.7). ΔCD47 H-scores were calculated as described in **c**.

Data are shown as the mean ± SD. ns, not significant. $^*P < 0.05$, $^{**}P < 0.01$, $^{***}P < 0.001$, unpaired two-tailed Student t test (**a**) or log-rank test (**b-d**). The experiment was performed three times with similar results (**a**). See Source Data file for the exact *P*-values. Source data are provided as a Source Data file.

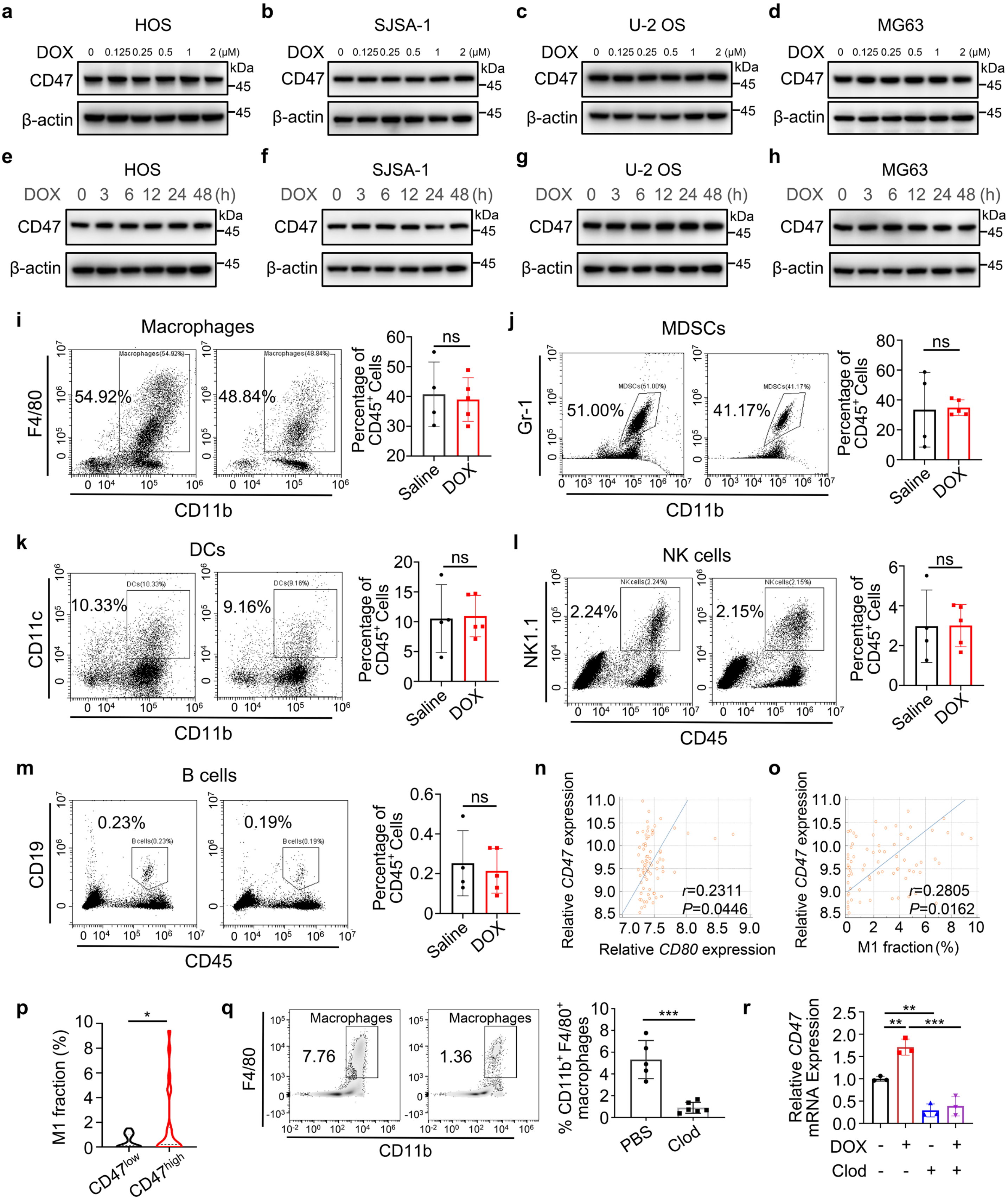


**Supplementary Figure 2, related to Fig. 2. Doxorubicin-induced CD47 upregulation in osteosarcoma is dependent on the activation of tumor associated macrophages.**

**a**-**d** Western blot analysis of CD47 expression in HOS (**a**), SJSA-1 (**b**), U-2 OS (**c**) and MG63 (**d**) cells treated with incremental doses of doxorubicin (DOX) for 24 h.

**e**-**h** Western blot analysis of CD47 expression in HOS (**e**), SJSA-1 (**f**), U-2 OS (**g**) and MG63 (**h**) cells treated with doxorubicin (DOX) for different time, as indicated.

**i**-**m** Flow cytometric analysis of CD11b$^+$ F4/80$^+$ macrophages (**i**), CD11b$^+$ Gr-1$^+$ myeloid-derived suppressor cells (MDSCs, **j**), CD11c$^+$ dendritic cells (DCs, **k**), NK1.1$^+$ nature killer (NK) cells (**l**), and CD19$^+$ B cells (**m**) among CD45$^+$ cells on day 22 in HOS tumors treated with saline or doxorubicin (DOX) every 2 days (*n*=4 mice for saline group and *n*=5 mice for doxorubicin group).

**n** Scatter plots showing the correlation between *CD47* and M1 macrophage marker *CD80* gene expression in osteosarcoma patients from GSE30699 (*n*= 76 patients).

**o, p** M1 macrophage fraction was calculated by CIBERSORT. **o** Scatter plots showing the correlation between *CD47* gene expression and M1 macrophage fraction of immune cells in osteosarcoma patients from GSE30699 (*n*= 73 patients). **p** Comparison of M1 macrophage fraction of immune cells between osteosarcoma patients with low (*n*=23) and high (*n*=23) *CD47* gene expression levels from the TARGET database. *CD47* gene expression levels were stratified based on a tertile method.

**q** Flow cytometric analysis of macrophages in HOS tumors treated with clodronate (Clod) liposome or PBS liposome on day 22 (*n*=5 mice PBS liposome group and *n*=6 mice for clodronate liposome group). Comparative analysis of the frequencies of CD11b$^+$ F4/80$^+$ macrophages in tumors is shown on the right.

**r** Quantitative real-time PCR analysis of *CD47* expression in saline- or doxorubicin (DOX)-treated HOS tumors administrated with clodronate (Clod) liposome or PBS liposome on day 22 (*n*=3 mice per group).

Data are shown as the mean ± SD. ns, not significant. $*P < 0.05$, $**P < 0.01$, $***P < 0.001$, unpaired two-tailed Student t test (**i**-**m**, **p**, **q**), Pearson correlation test (**n, o**) or one-way ANOVA (**r**). The experiment was performed three times with similar results (**a**-**m**, **q**, **r**). See Source Data file for the exact *P*-values. Source data are provided as a Source Data file.

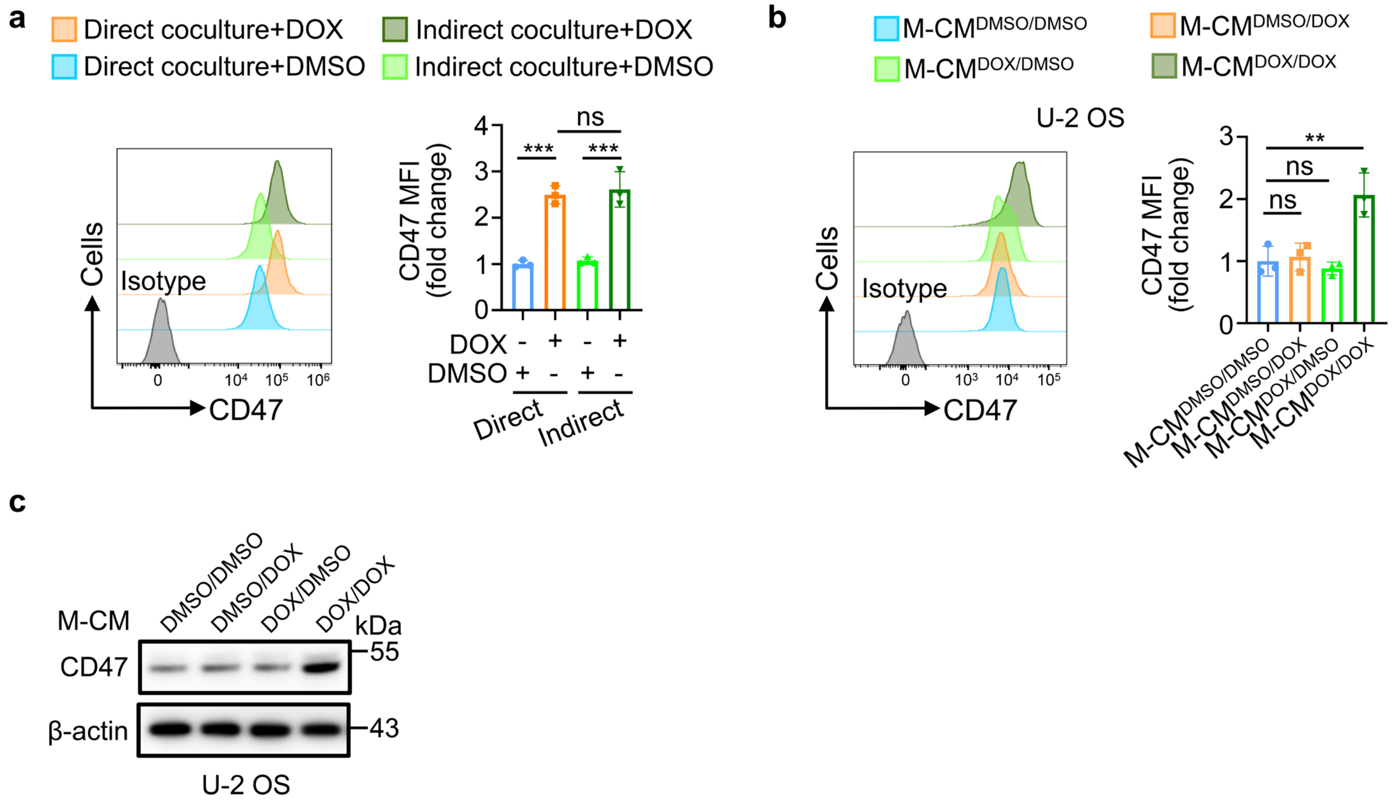


**Supplementary Figure 3, related to Fig. 3. Macrophage-secreted factors induced by doxorubicin promote CD47 expression in osteosarcoma cells for inhibition of macrophages phagocytosis.**

**a** HOS tumor cells were cocultured with THP-1-derived macrophages in the presence of DMSO or doxorubicin (DOX) for 48 h. Direct coculture where HOS cells and macrophages were seeded in the same culture well. Indirect coculture where HOS cells and macrophages were separated from each other with transwell inserts. CD47 expression on the tumor cell surface was determined by flow cytometry using isotype control or anti-CD47 antibody (left). GFP positive events that represent HOS cells were gated for CD47 analysis and the anti-CD47 median fluorescence intensity (MFI) was determined (right). *n*=3 independent experiments.

**b**, **c** U-2 OS cells were treated with M-CM$^{DMSO/DMSO}$, M-CM$^{DMSO/DOX}$, M-CM$^{DOX/DMSO}$, or M-CM$^{DOX/DOX}$ for 24 h. **b** CD47 expression on the cell surface was determined by flow cytometry using isotype control or anti-CD47 antibody (left). The anti-CD47 median fluorescence intensity (MFI) was determined (right). *n*=3 independent experiments. **c** Western blot analysis of CD47 expression in U-2 OS cells.

Data are shown as the mean ± SD. ns, not significant. $**P < 0.01$, $***P < 0.001$, one-way ANOVA (**a**, **b**). The experiment was performed three times with similar results (**c**). See Source Data file for the exact *P*-values. Source data are provided as a Source Data file.

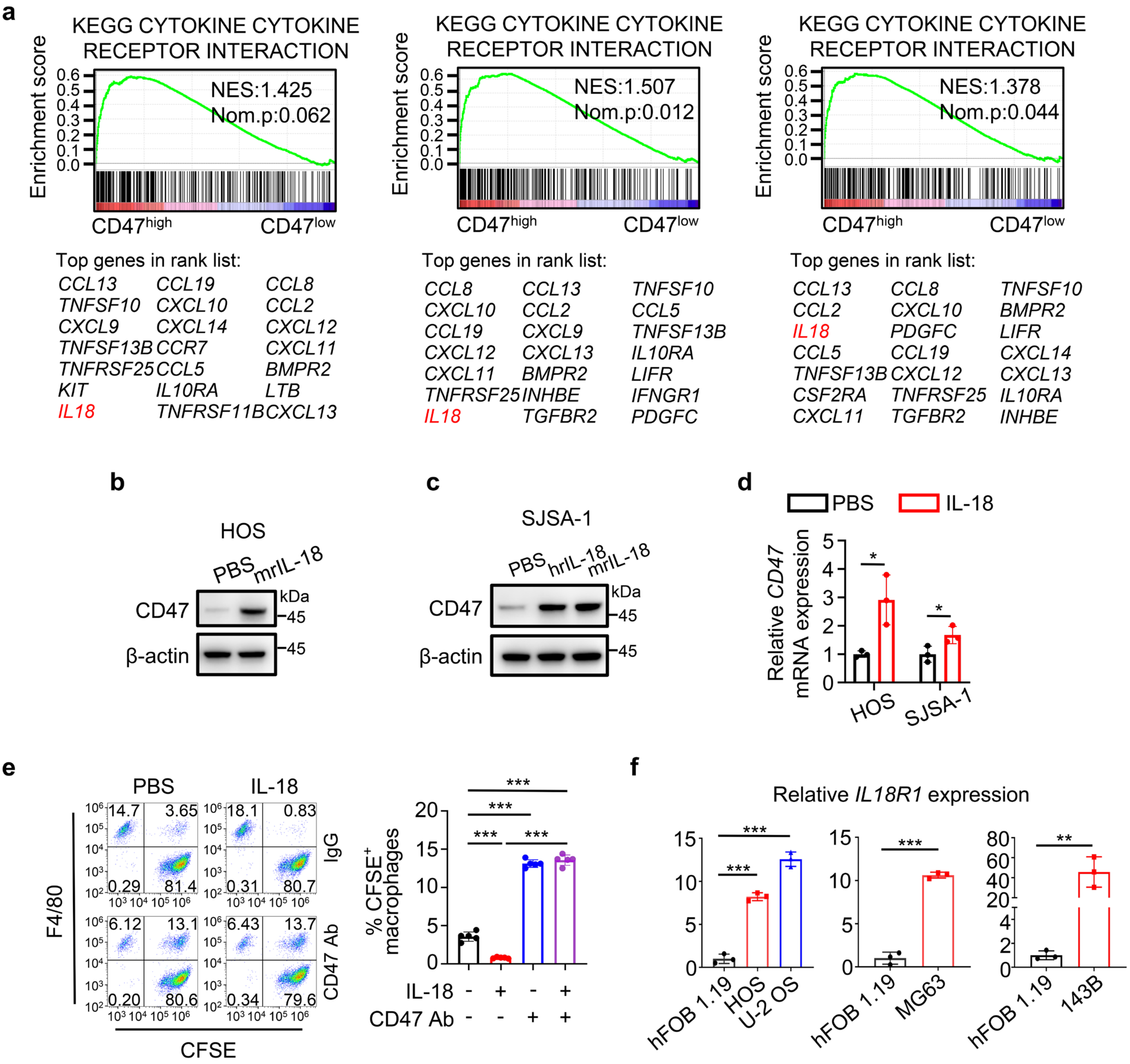


**Supplementary Figure 4, related to Fig. 4. Macrophage-secreted IL-18 induces CD47 expression in osteosarcoma cells.**

**a** GSEA plots of the “cytokine-cytokine receptor interaction” pathway compared between osteosarcoma patients with high ($n$=19) and low ($n$=19) levels of *CD47* expression from dataset GSE30699 (left), between osteosarcoma patients with high ($n$=14) and low ($n$=13) levels of *CD47* expression from dataset GSE21257 (middle), and between osteosarcoma patients with high ($n$=21) and

low (*n*=21) levels of *CD47* expression from dataset GSE33382 (right). *CD47* gene expression was stratified based on the quartile method. Top genes in ranked lists are shown below the plots.

**b** Western blot analysis of HOS cells treated with PBS or mouse recombinant (mr) IL-18 (40 ng/ml) for 24 h.

**c** Western blot analysis of SJSA-1 cells treated with PBS, human recombinant (hr) IL-18 (40 ng/ml) or mouse recombinant (mr) IL-18 (40 ng/ml) for 24 h.

**d** Quantitative real-time PCR analysis of *CD47* expression in HOS and SJSA-1 cells treated with PBS or IL-18 (40 ng/ml) for 24 h (*n*=3 independent experiments).

**e** Flow cytometry-based in vitro macrophage-mediated phagocytosis assay of SJSA-1 cells in the presence of IgG or anti-CD47 antibody for 4h (*n*=5 independent experiments). SJSA-1 cells were pretreated with PBS or IL-18 (40 ng/ml) for 24 h. Macrophages were defined as F4/80$^+$ (labeled with PE) events, and tumor cells as CSFE$^+$ (labeled with CFSE). F4/80$^+$, CFSE$^+$ events represented macrophages that had phagocytosed tumor cells.

**f** Quantitative real-time PCR analysis of *IL18R1* expression in the osteoblast cell line, hFOB 1.19, and the osteosarcoma cell lines, HOS, U-2 OS, MG63, and 143B (*n*=3 independent experiments).

Data are shown as the mean ± SD. ns, not significant. **P* < 0.05, ***P* < 0.01, ****P* < 0.001, unpaired two-tailed Student t test (**d**, **f**), or one-way ANOVA (**e**). The experiment was performed three times with similar results (**b**, **c**). See Source Data file for the exact *P*-values. Source data are provided as a Source Data file.

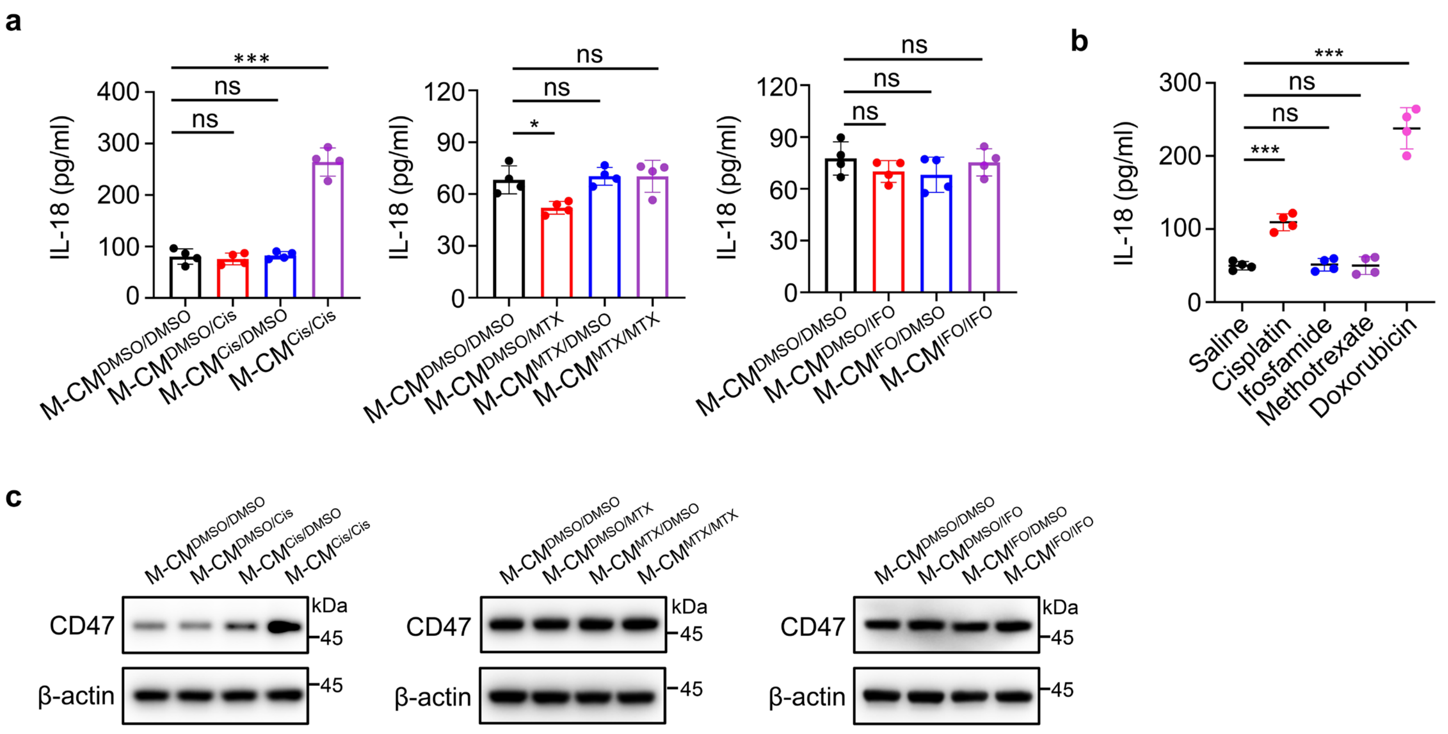


**Supplementary Figure 5. Cisplatin, but not methotrexate or ifosfamide, induces IL-18 secretion by macrophages and CD47 upregulation on tumor cells.**

**a** Cisplatin (Cis; 10 µM; Selleck Chemicals), methotrexate (MTX; 15 µM; Selleck Chemicals) or ifosfamide (IFO; 15 µM; Selleck Chemicals) was used to produce four kinds of macrophage conditioned media (M-CM) as indicated in **Fig. 3a**. During the production process, HOS cells were treated with these drugs for 24h. Macrophages were treated with these drugs in the presence of T-CM$^{DMSO}$ or T-CM$^{DOX}$ for 12 h. Elisa analysis of IL-18 concentrations in four kinds of M-CM (*n*=4 independent experiments).

**b** ELISA analysis of IL-18 concentrations in HOS tumor tissues treated with saline, cisplatin, ifosfamide, methotrexate, or doxorubicin on day 22 (*n*=4 mice per group).

**c** Western blot analysis of CD47 expression in HOS cells treated with four kinds of M-CM produced as indicated in **a** for 24 h.

Data are shown as the mean ± SD. ns, not significant. $*P < 0.05$, $***P < 0.001$, one-way ANOVA (**a**, **b**). The experiment was performed three times with similar results (**b**, **c**). See Source Data file for the exact *P*-values. Source data are provided as a Source Data file.

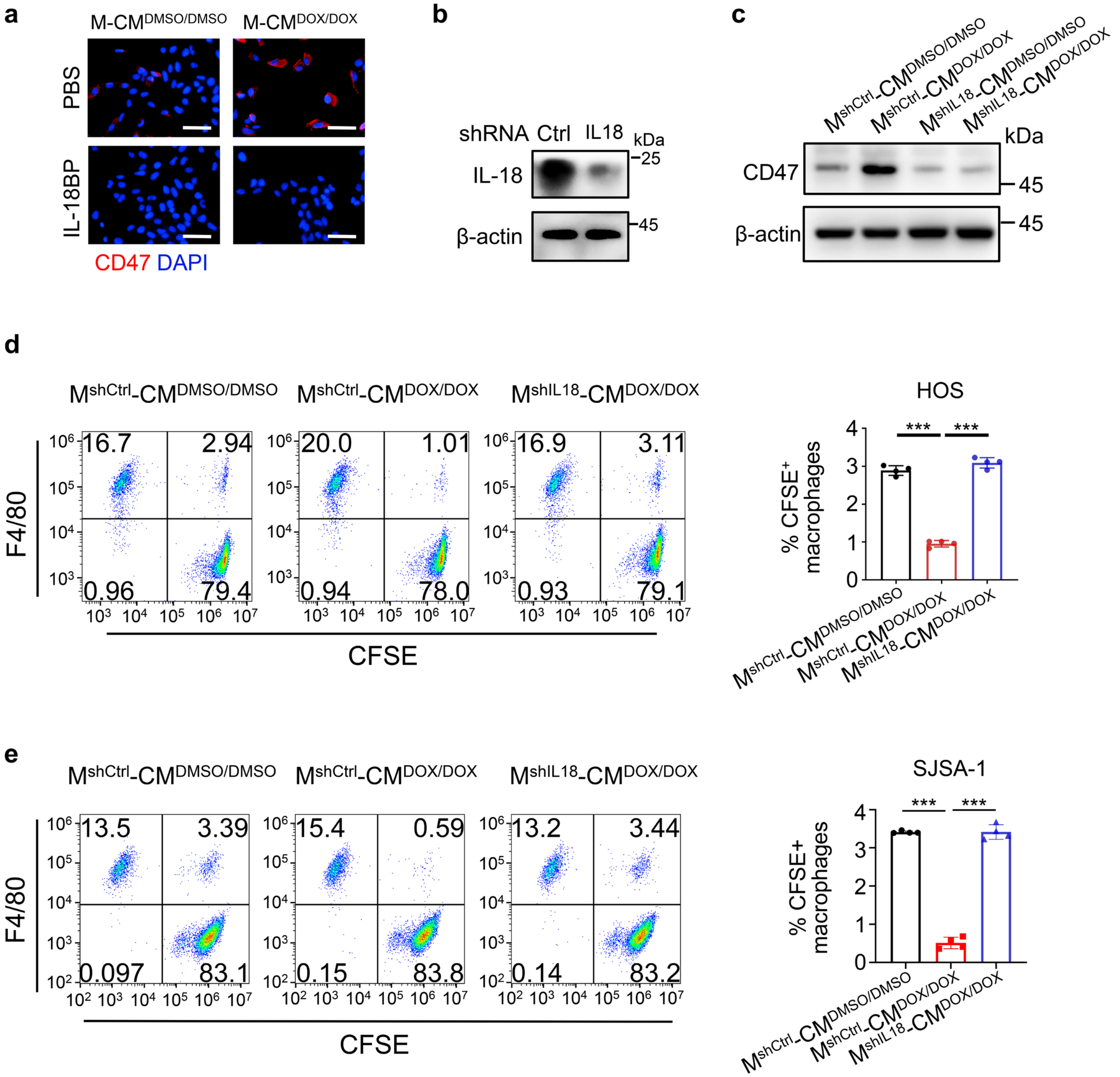


**Supplementary Figure 6. IL-18 blockade reduces CD47 expression on tumor cells and enhances macrophage phagocytosis of tumor cells.**

**a** Immunofluorescence staining of CD47 in HOS cells. HOS cells were treated with M-CM$^{DMSO/DMSO}$ or M-CM$^{DOX/DOX}$ in the presence or absence of IL-18BP (2.5 μg/ml) for 24 h. Scale bar, 150 μm.

**b** Western blot analysis of IL-18 expression in shCtrl or shIL18 THP-1 cells.

**c-e** THP-1 cells with or without depletion of IL18 ($M^{shCtrl}$ or $M^{shIL18}$) were used to produce macrophage conditioned media as described in **Fig. 3a**. $M^{shCtrl}$-$CM^{DMSO/DMSO}$ was produced from $M^{shCtrl}$ with DMSO, $M^{shCtrl}$-$CM^{DOX/DOX}$ was produced from $M^{shCtrl}$ with doxorubicin (DOX), $M^{shIL18}$-$CM^{DMSO/DMSO}$ was produced from $M^{shIL18}$ with DMSO, and $M^{shIL18}$-$CM^{DOX/DOX}$ was produced from $M^{shIL18}$ with DOX. **c** Western blot analysis of HOS cells treated as indicated for 24 h. **d, e** Flow cytometry-based in vitro macrophage phagocytosis assay of HOS (**d**) or SJSA-1 (**e**) cells treated as indicated for 24 h ($n$=4 independent experiments). Macrophages were defined as F4/80$^+$ (labeled with PE) events, and tumor cells as CFSE$^+$ events (stained with CFSE). F4/80$^+$, CFSE$^+$ events represented macrophages that had phagocytosed tumor cells.

Data are shown as the mean ± SD. ***$P$ < 0.001, one-way ANOVA (**d**, **e**). The experiment was performed three times with similar results (**a-c**). See Source Data file for the exact $P$-values. Source data are provided as a Source Data file.

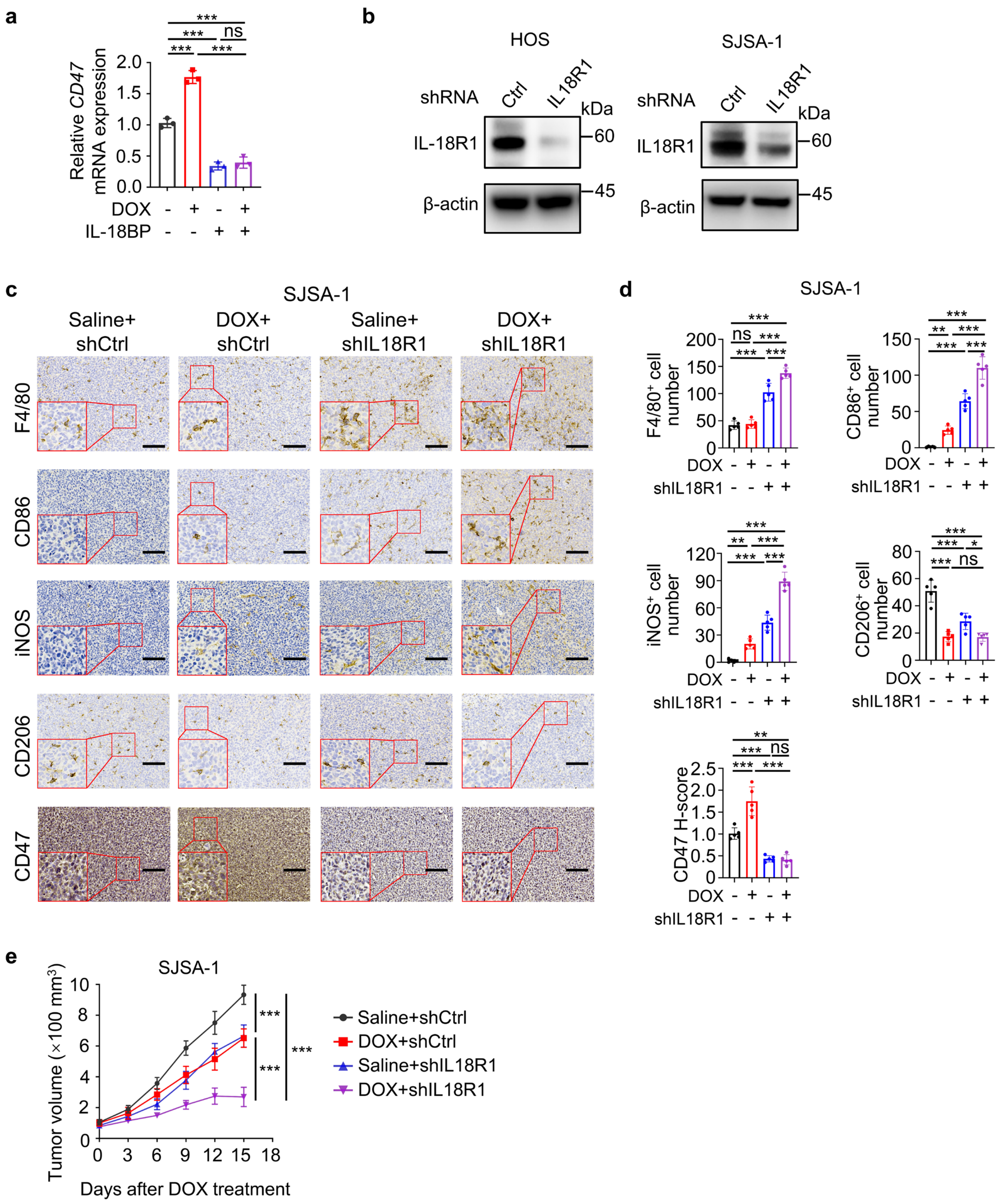


**Supplementary Figure 7, related to Fig. 5. IL-18 blockade sensitizes osteosarcoma to doxorubicin treatment.**

**a** Quantitative real-time PCR analysis of *CD47* expression in tumors treated with saline or doxorubicin (DOX), with or without mouse recombinant (mr) IL-18BP on day 22 (*n*=3 mice per group).

**b** Western blot analysis of IL-18R1 expression in shCtrl or shIL18R1 HOS (left) or SJSA-1 (right) cells.

**c-e,** Mice bearing SJSA-1 tumors with or without IL18R1 depletion were treated with saline or doxorubicin (DOX) after tumors grew for 14 days. **c** Representative immunohistochemical images, showing F4/80, CD86, iNOS, CD206 and CD47 expression in serial sections of SJSA-1 tumors on day 29. Scale bar, 300 μm. **d** Statistical analyses of quantification of **c** (*n*=5 mice per group). **e** Tumor growth of SJSA-1 cells in mice (*n*=5 mice per group). Tumor volume was measured at the indicated time points.

Data are shown as the mean ± SD. ns, not significant. **P*< 0.05, ***P* < 0.01, ****P* < 0.001, one-way ANOVA (**a**, **d**) or two-way ANOVA (**e**). The experiment was performed three times with similar results (**a-e**). See Source Data file for the exact *P*-values. Source data are provided as a Source Data file.

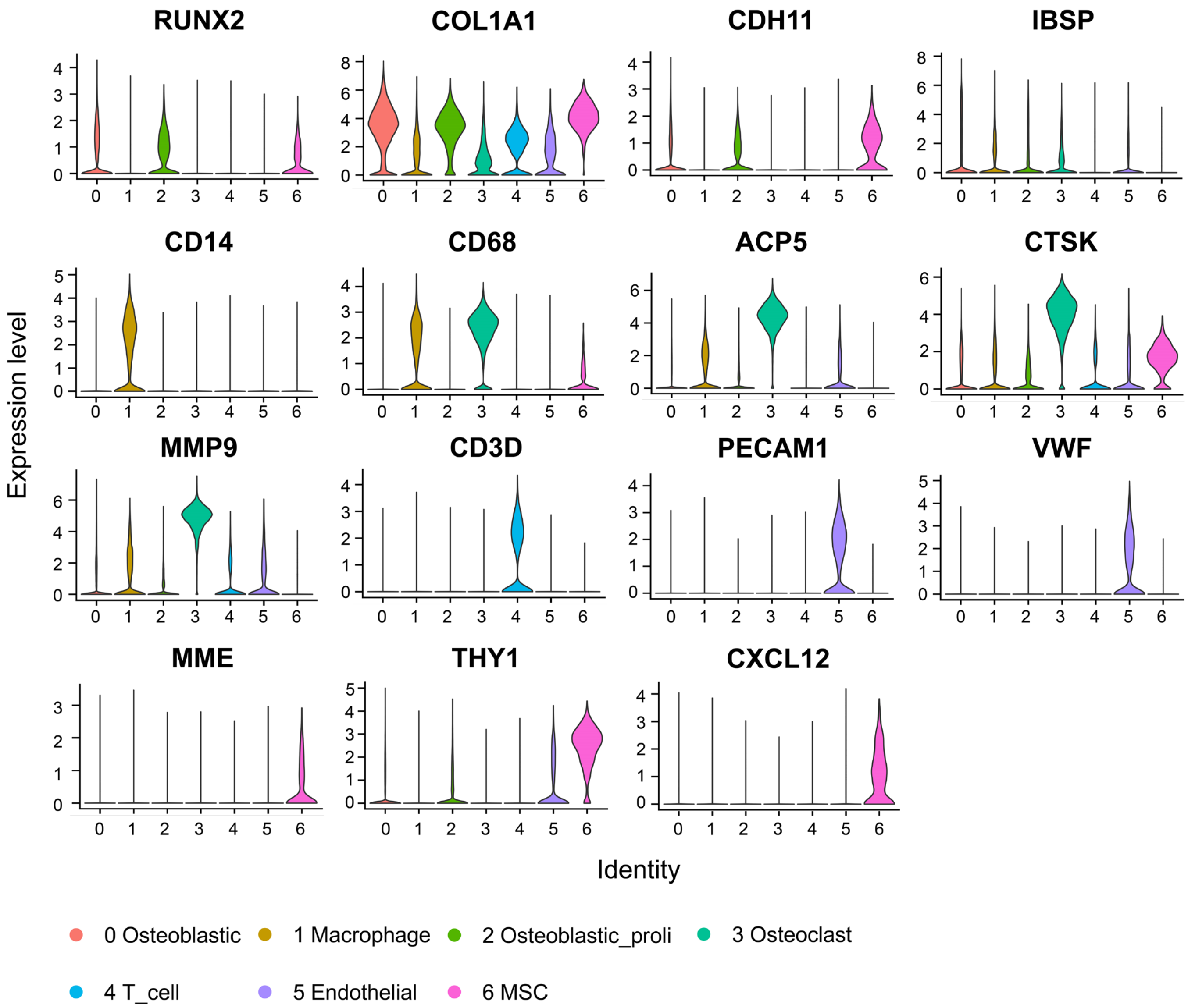


**Supplementary Figure 8. Expression level of canonical biomarkers in each subcluster.** The violin plot showing the normalized expression levels of 15 specific canonical genes for each of the 7 indicated clusters ($n$=11 osteosarcoma samples of patients).

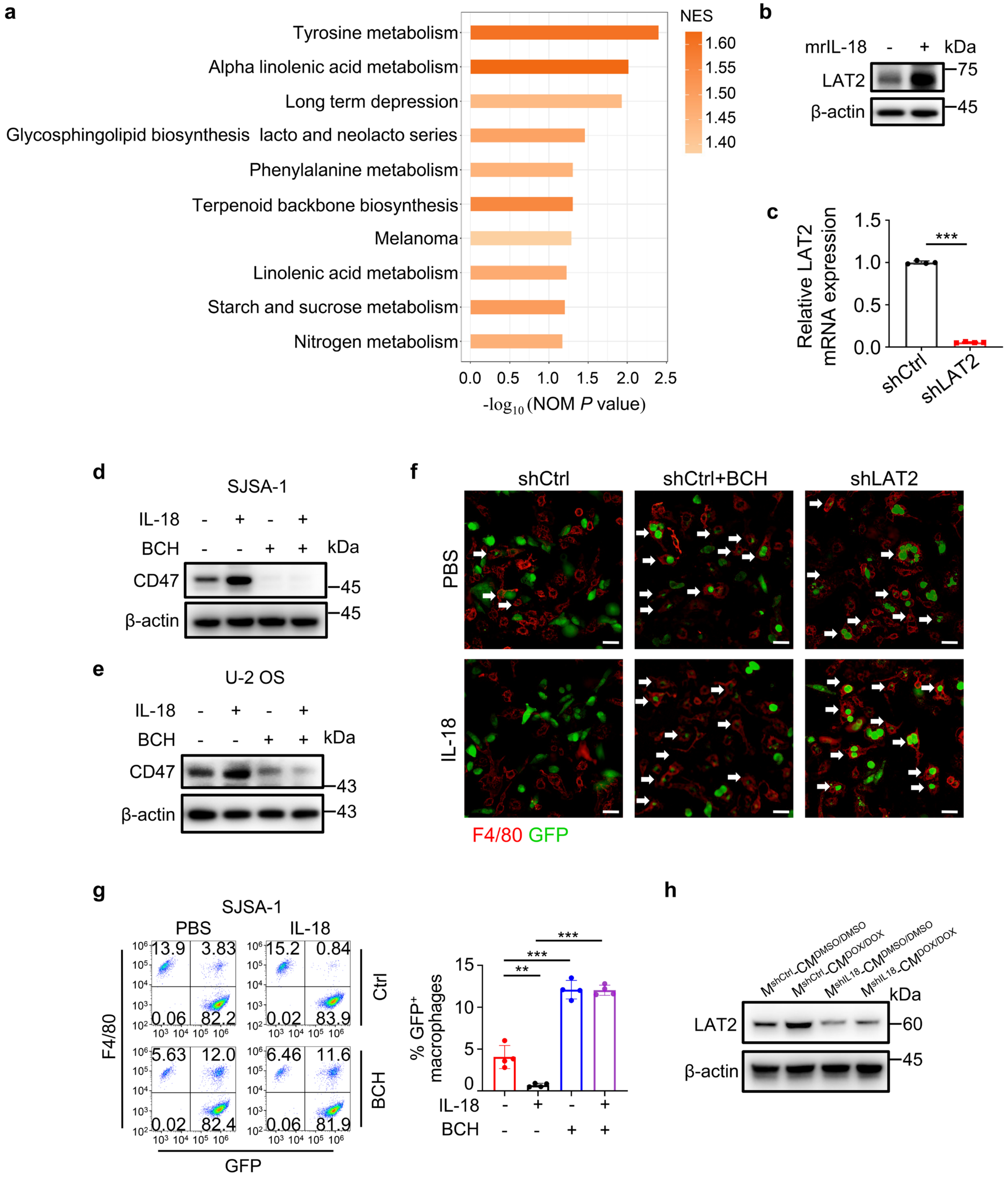


**Supplementary Figure 9, related to Fig. 7. Amino acid transporter LAT2-mediated amino acid uptake is necessary for IL-18-induced CD47 expression in osteosarcoma cells.**

**a** KEGG pathways enriched in osteosarcoma patients with higher CD47 expression from TARGET database analyzed by GSEA. GSEA was performed between patients with higher (*n*=43) or lower (*n*=43) CD47 expression. *CD47* gene expression was stratified based on the median method.

**b** Western blot analysis of LAT2 expression in HOS cells following mouse recombinant (mr) IL-18 (40 ng/ml) or PBS treatment for 24 h.

**c** Quantitative real-time PCR analysis of *LAT2* expression in shLAT2 or shCtrl HOS cells (*n*=4 independent experiments).

**d, e** Western blot analysis of SJSA-1 (**d**) or U-2 OS (**e**) cells treated with PBS or IL-18 in the presence or absence of BCH, for 24 h.

**f** Fluorescence microscopy images showing macrophage phagocytosis of shCtrl HOS cells, BCH-treated shCtrl HOS cells, or shLAT2 HOS cells, treated with PBS or IL-18. Macrophages were defined as cells staining with anti-F4/80-PE antibody (red) and HOS cells by GFP expression (green). Scale bar, 150 μm.

**g** Flow cytometry-based analysis of macrophage phagocytosis of SJSA-1 cells treated with or without BCH (*n*=4 independent experiments). Macrophages were defined as F4/80$^+$ (labeled with PE) events, and tumor cells as GFP$^+$ events. F4/80$^+$, GFP$^+$ events represented macrophages that had phagocytosed tumor cells.

**h** THP-1 cells with or without depletion of IL18 ($M^{shCtrl}$ or $M^{shIL18}$) were used to produce macrophage conditioned media (M-CM) as described in **Fig. 3a**. $M^{shCtrl}$-$CM^{DMSO/DMSO}$ was produced from $M^{shCtrl}$ with DMSO, $M^{shCtrl}$-$CM^{DOX/DOX}$ was produced from $M^{shCtrl}$ with doxorubicin (DOX), $M^{shIL18}$-$CM^{DMSO/DMSO}$ was produced from $M^{shIL18}$ with DMSO, and $M^{shIL18}$-$CM^{DOX/DOX}$ was produced from $M^{shIL18}$ with DOX. Western blot analysis of LAT2 expression in HOS cells treated with M-CM for 24 h.

Data are shown as the mean ± SD. $^{**}P < 0.01$, $^{***}P < 0.001$, unpaired two-tailed Student t test (**c**) or one-way ANOVA (**g**). The experiment was performed three times with similar results (**b**, **d-f**, **h**). See Source Data file for the exact *P*-values. Source data are provided as a Source Data file.

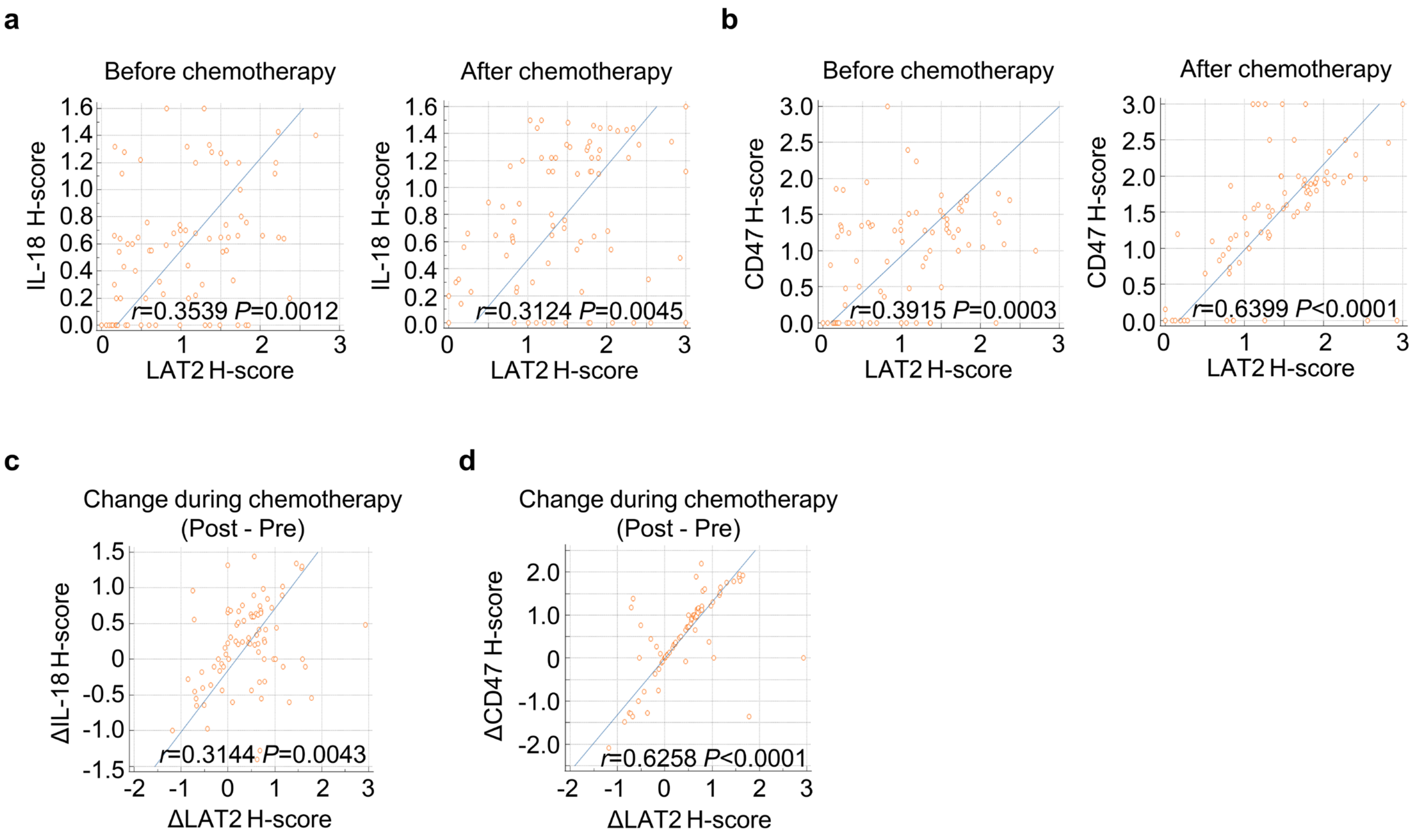


**Supplementary Figure 10. LAT2 expression positively correlates with IL-18/CD47 expression in osteosarcoma patients.**

**a** Scatter plots showing the correlation between IL-18 and LAT2 expression in pre-chemotherapy (left) or post-chemotherapy (right) specimens of osteosarcoma patients ($n$=81 patients).

**b** Scatter plots showing the correlation between CD47 and LAT2 expression in pre-chemotherapy (left) or post-chemotherapy (right) specimens of osteosarcoma patients ($n$=81 patients).

**c** Scatter plots showing the correlation between changes of IL-18 and LAT2 expression during chemotherapy. ΔIL-18/ΔLAT2 H-score for each patient was obtained from subtracting IL-18/LAT2 H-score of pre-chemotherapy specimens from that of paired post-chemotherapy specimens ($n$=81 patients).

**d** Scatter plots showing the correlation between changes of CD47 and LAT2 expression during chemotherapy. ΔCD47/ΔLAT2 H-score for each patient was obtained from subtracting ΔCD47/Δ LAT2 H-score of pre-chemotherapy specimens from that of paired post-chemotherapy specimens ($n$=81 patients).

Pearson correlation test (**a-d**). Source data are provided as a Source Data file.

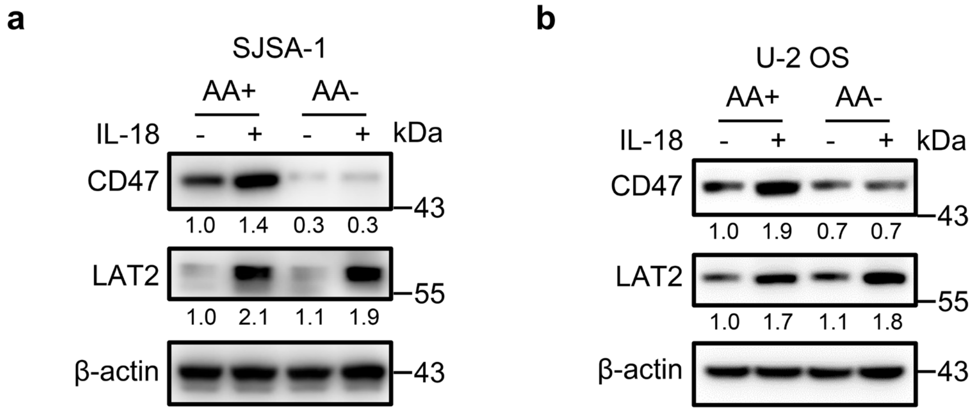


**Supplementary Figure 11. BCH treatment diminishes IL-18-induced CD47 expression on tumor cells.**

**a, b** Western blot analysis of amino acid-starved SJSA-1 (**a**) or U-2 OS (**b**) cells treated with IL-18 or PBS in amino acid-free (AA-) or amino acid-sufficient (AA+) medium for 21 h. The experiment was performed three times with similar results (**a**, **b**).

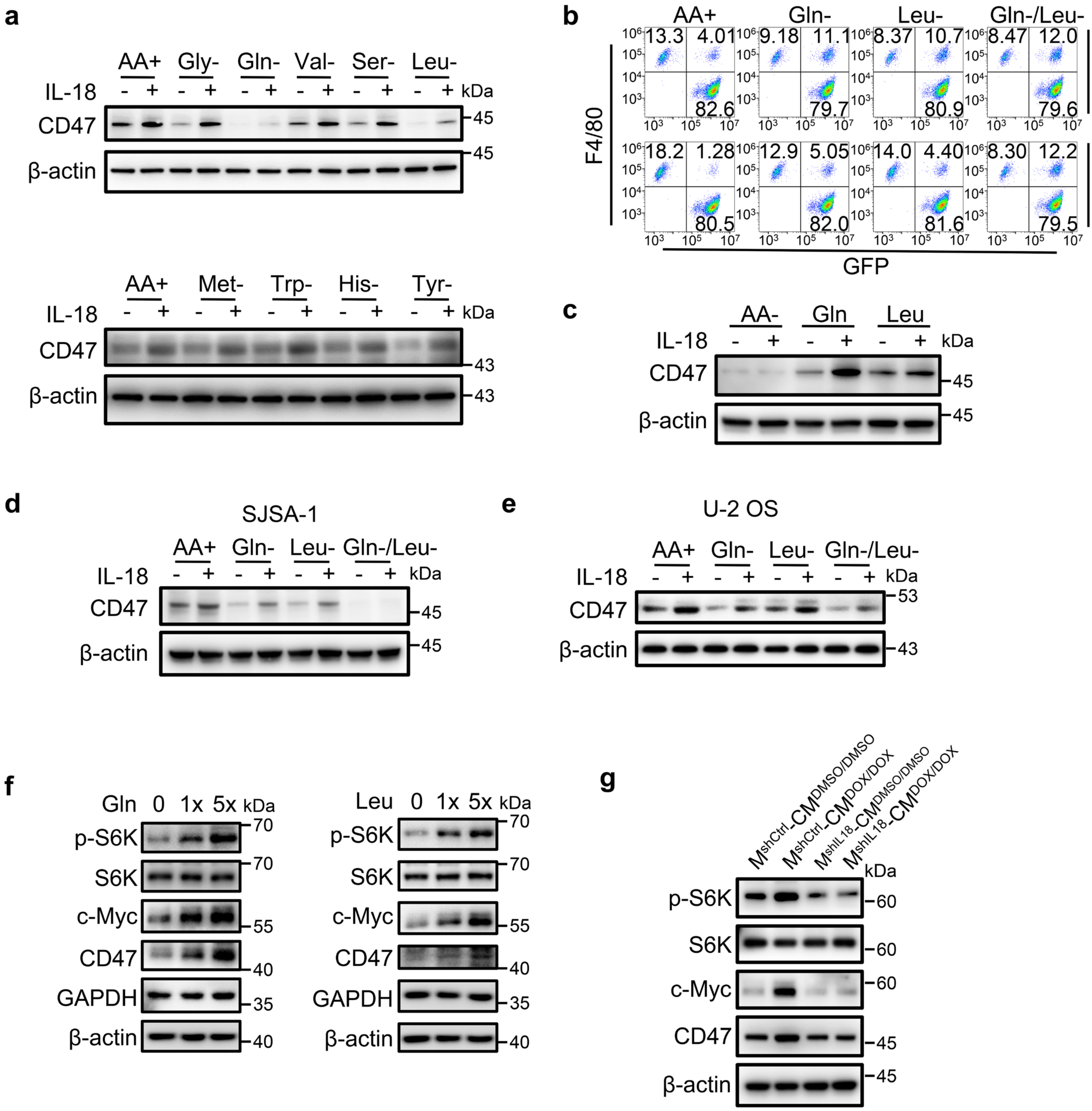


**Supplementary Figure 12, related to Fig. 8. IL-18-enhanced and LAT2-mediated uptake of glutamine and leucine promotes CD47 expression by activating mTORC1/c-Myc axis.**

**a** Western blot analysis of HOS cells treated with or without IL-18 (40 ng/ml) in amino acid-sufficient medium (AA+) or medium lacking indicated amino acids for 24 h. Gly-, medium lacking glycine; Gln-, medium lacking glutamine; Val-, medium lacking valine; Ser-, medium lacking serine; Leu-, medium lacking leucine; Met-, medium lacking methionine; Trp-, medium lacking tryptophan; His-, medium

lacking histidine; Tyr-, medium lacking tyrosine.

**b** Representative flow plots of **Fig. 8b**.

**c** Western blot analysis of HOS cells that were rested in amino-acid free (AA-) medium for 3 h and then restimulated with indicated amino acids for 21 h, in the presence or absence of IL-18 stimulation. Gln, glutamine; Leu, leucine.

**d**, **e** Western blot analysis of SJSA-1 (**d**) and U-2 OS (**e**) cells treated with PBS or IL-18 (40 ng/ml) in amino acid-sufficient medium (AA+) or medium lacking indicated amino acids for 24 h. Gln-, medium lacking glutamine; Leu-, medium lacking leucine; Gln-/Leu-, medium lacking glutamine and leucine.

**f** Western blot analysis of amino acid-starved HOS cells incubated with amino acid free medium (AA-) or medium only containing glutamine (Gln, left) or leucine (Leu, right) for 21 h. 1×, the concentration of indicated amino acid was the same as that in commercially-available DMEM. 5×, the concentration of indicated amino acid was five times that in commercially-available DMEM.

**g** THP-1 cells with or without depletion of IL18 ($M^{shCtrl}$ or $M^{shIL18}$) were used to produce macrophage conditioned media (M-CM) as described in **Fig. 3a**. $M^{shCtrl}$-$CM^{DMSO/DMSO}$ was produced from $M^{shCtrl}$ with DMSO, $M^{shCtrl}$-$CM^{DOX/DOX}$ was produced from $M^{shCtrl}$ with doxorubicin (DOX), $M^{shIL18}$-$CM^{DMSO/DMSO}$ was produced from $M^{shIL18}$ with DMSO, and $M^{shIL18}$-$CM^{DOX/DOX}$ was produced from $M^{shIL18}$ with DOX. Western blot analysis of HOS cells treated with M-CM for 24 h.

The experiment was performed three times with similar results (**a**, **c-g**).

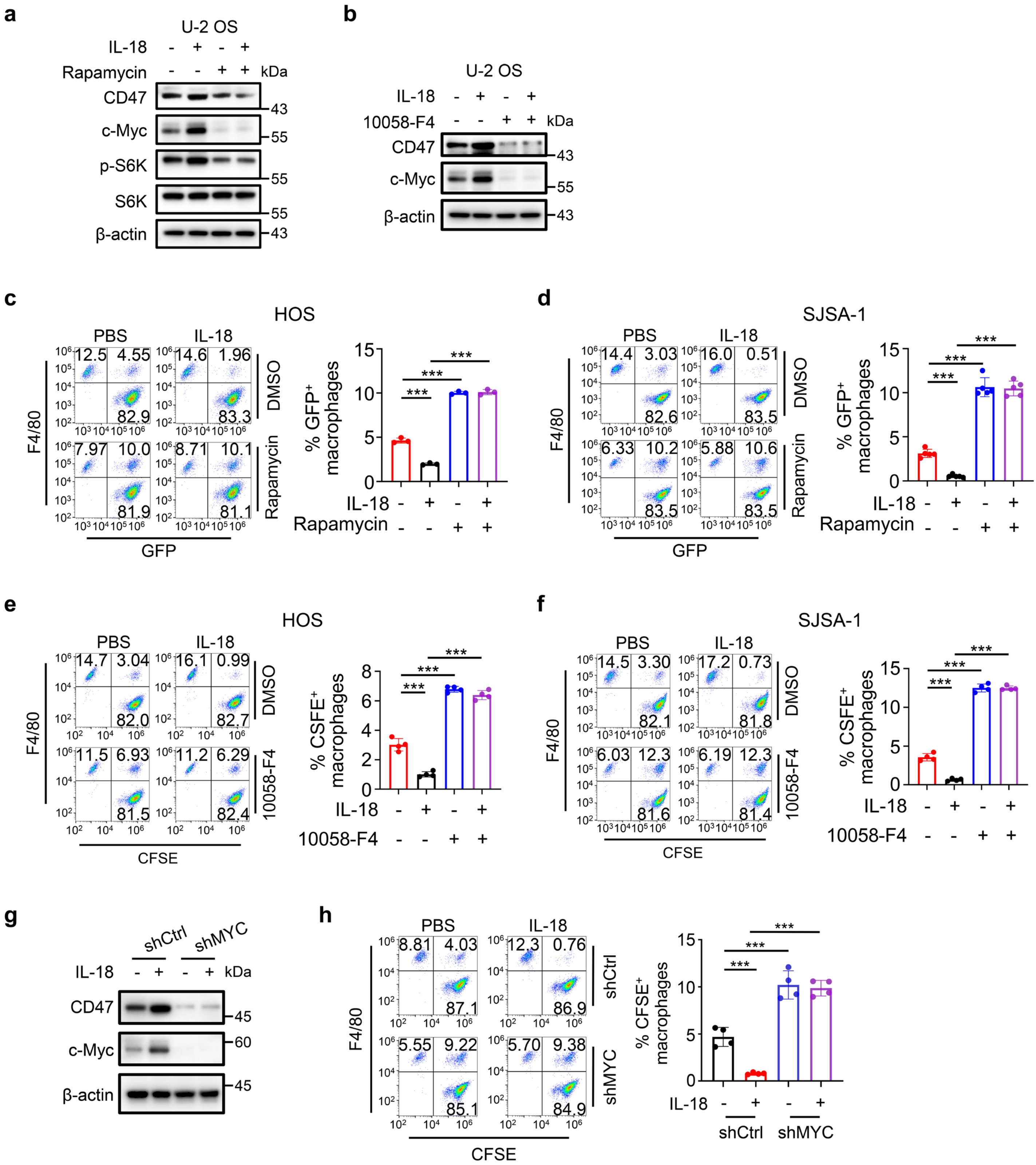


**Supplementary Figure 13. Rapamycin or c-MYC blockade abolishes IL-18-induced CD47 expression and IL-18-supressed macrophages phagocytosis.**

**a** Western blot analysis of U-2 OS cells treated with PBS or IL-18 (40 ng/ml) in the presence or absence of rapamycin (25 nM) for 24 h. Cells were pre-treated with Rapamycin for 1 h before the addition of IL-18.

**b** Western blot analysis of U-2 OS cells treated with PBS or IL-18 (40 ng/ml) in the presence or absence of 10058-F4 (100 μM) for 24 h. Cells were pre-treated with 10058-F4 for 1 h before the addition of IL-18.

**c, d** Flow cytometry-based in vitro macrophage phagocytosis assay. HOS (**c**) or SJSA-1 (**d**) cells were treated with PBS or IL-18 (40 ng/ml) in the presence or absence of rapamycin (25 nM) for 24 h ($n$=3 independent experiments for HOS cells and $n$=5 independent experiments for SJSA-1 cells). Macrophages were defined as F4/80$^+$ (labeled with PE) events, and tumor cells as GFP$^+$ events. F4/80$^+$, GFP$^+$ events represented macrophages that had phagocytosed tumor cells.

**e**, **f** Flow cytometry-based in vitro macrophage phagocytosis assay. HOS (**e**) or SJSA-1 (**f**) cells were treated with PBS or IL-18 (40 ng/ml) in the presence or absence of 10058-F4 (100 μM) for 24 h ($n$=4 independent experiments). Macrophages were defined as F4/80$^+$ (labeled with PE) events, and tumor cells as CFSE$^+$ (labeled with CFSE) events. F4/80$^+$, CFSE$^+$ events represented macrophages that had phagocytosed tumor cells.

**g**, **h** shCtrl or shMYC HOS cells were treated with PBS or IL-18 (40 ng/ml) for 24 h. **g** Western blot analysis of HOS cells. **h** Flow cytometry-based in vitro macrophage phagocytosis assay of HOS cells ($n$=4 independent experiments). Macrophages were defined as F4/80$^+$ (labeled with PE) events, and tumor cells as CFSE$^+$ (labeled with CFSE) events. F4/80$^+$, CFSE$^+$ events represented macrophages that had phagocytosed tumor cells.

Data are shown as the mean ± SD. ***$P$ < 0.001, one-way ANOVA (**c-f, h**). The experiment was performed three times with similar results (**a**, **b, g**). See Source Data file for the exact $P$-values. Source data are provided as a Source Data file.

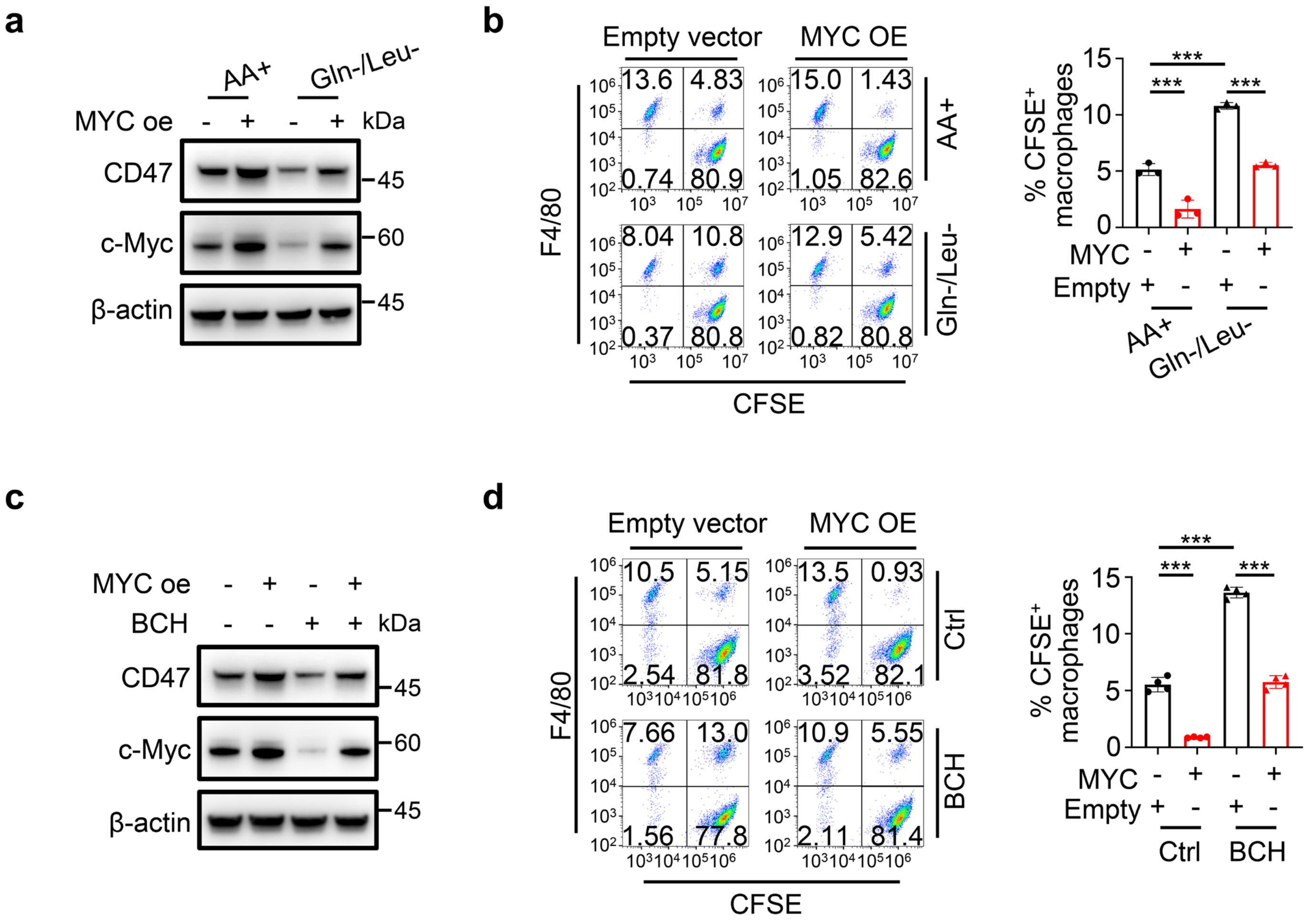


**Supplementary Figure 14. c-Myc overexpression bypasses the Glu/Leu deprivation- and BCH treatment-dependent regulation.**

**a, b** HOS cells infected with lentivirus expressing empty vector or MYC were cultured in amino acid-sufficient medium (AA+) or medium lacking glutamine and leucine (Gln-/Leu-) for 24 h. **a** Western blot analysis of HOS cells treated as described. **b** Flow cytometry-based in vitro macrophage phagocytosis assay of HOS cells treated as described (*n*=3 independent experiments). Macrophages were defined as F4/80$^+$ (labeled with PE) events, and tumor cells as CFSE$^+$ (labeled with CFSE) events. F4/80$^+$, CFSE$^+$ events represented macrophages that had phagocytosed tumor cells.

**c**, **d** HOS cells infected with lentivirus expressing empty vector or MYC were treated with or without BCH for 24 h. **c** Western blot analysis of HOS cells treated as described. **d** Flow cytometry-based in vitro macrophage phagocytosis assay of HOS cells treated as described (*n*=4 independent experiments). Macrophages were defined as F4/80$^+$ (labeled with PE) events, and tumor cells as CFSE$^+$ (labeled with CFSE) events. F4/80$^+$, CFSE$^+$ events represented macrophages that had phagocytosed tumor cells.

Data are shown as the mean ± SD. ***$P < 0.001$, one-way ANOVA (**b, d**). The experiment was performed three times with similar results (**a, c**). See Source Data file for the exact *P*-values. Source data are provided as a Source Data file.

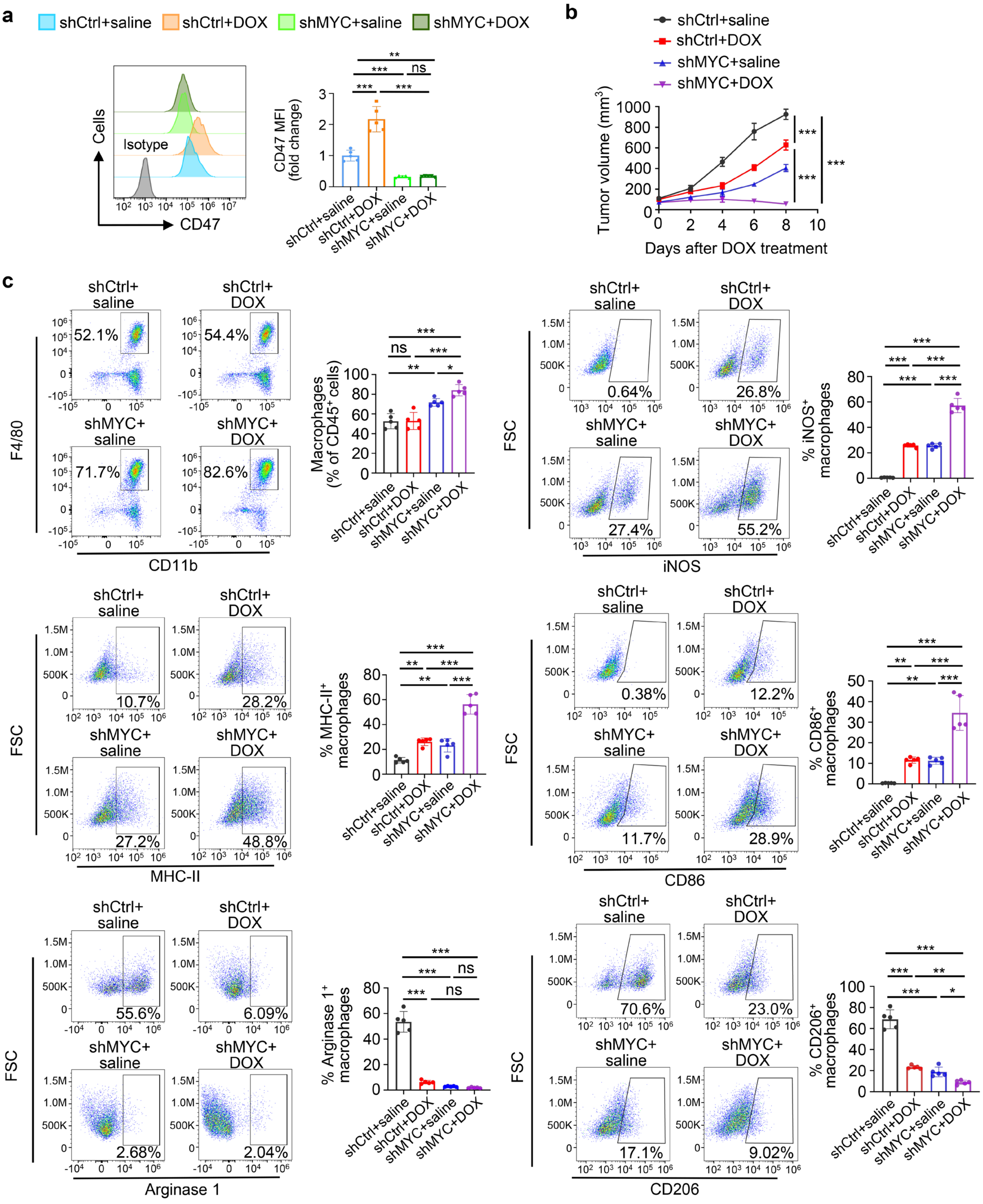


**Supplementary Figure 15. MYC depletion sensitizes osteosarcoma to doxorubicin treatment.**

**a-c** Mice were treated with saline or doxorubicin (DOX) every 2 days after shCtrl or shMYC HOS tumors grew for 14 days. **a** Flow cytometric analysis of CD47 expression in HOS cells from tumors on day 22 (left; *n*=5 mice per group). HOS cells were additionally labeled with GFP and $GFP^+$ events that represent HOS cells were gated for CD47 analysis. The anti-CD47 median fluorescence intensity (MFI) was determined (right). **b**Tumor volume measured at the indicated time points (*n*=5 mice per group). **c** Flow cytometric analysis of MHC-II, iNOS, CD86, Arginase 1, and CD206 expression in $CD45^+$ $CD11b^+$ $F4/80^+$ macrophages in tumors on day 17 (*n*=5 mice per group).

Data are shown as the mean ± SD. ns, not significant. *$P < 0.05$, **$P < 0.01$, ***$P < 0.001$, one-way ANOVA (**a**, **c**) or two-way ANOVA (**b**). The experiment was performed twice (**a-c**) with similar results. See Source Data file for the exact *P*-values. Source data are provided as a Source Data file.

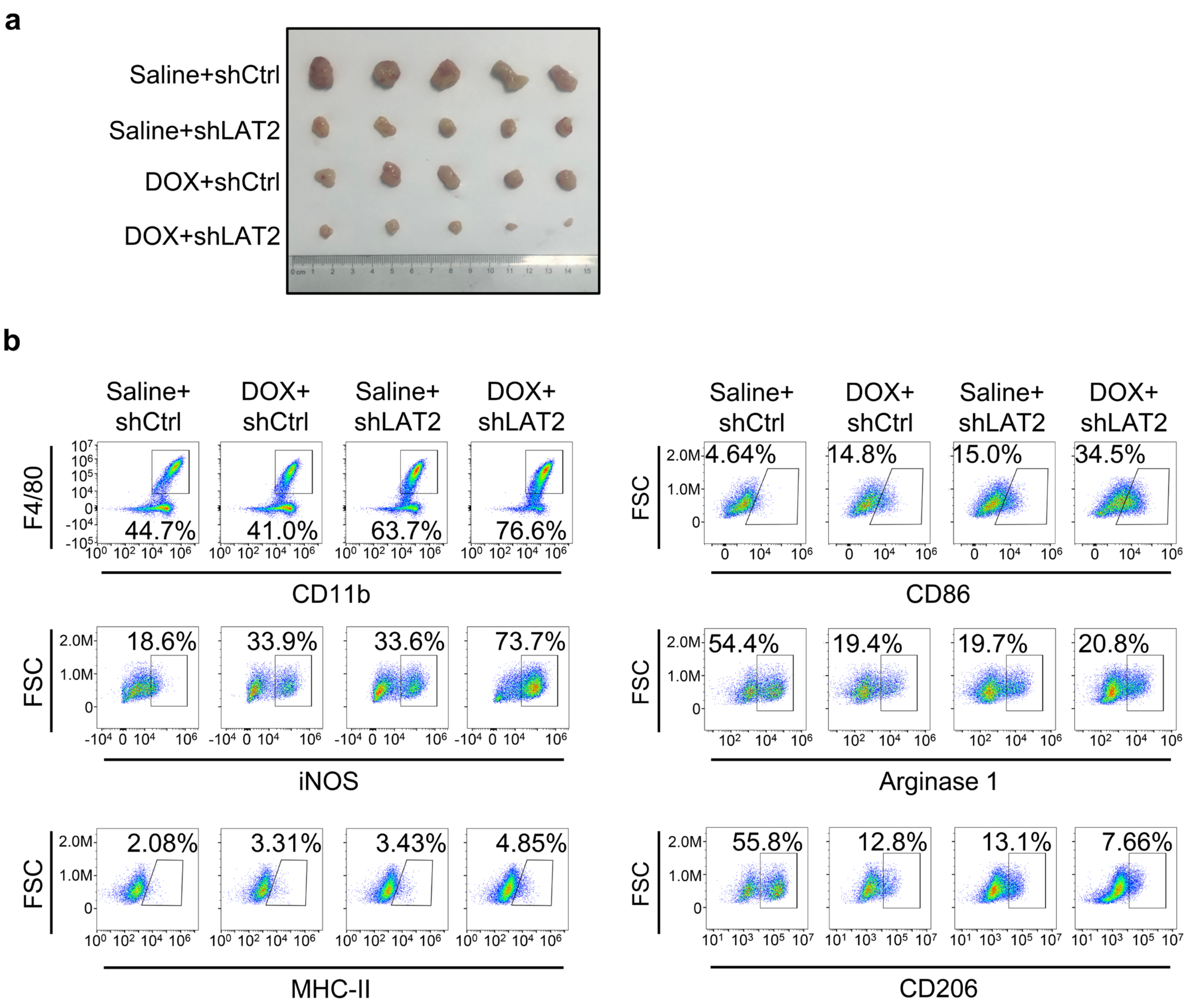


**Supplementary Figure 16, related to Fig. 9. LAT2 depletion sensitizes osteosarcoma to doxorubicin treatment.**

**a** Representative images of HOS tumors treated as described in **Fig. 9a-d** on day 22.

**b** Representative flow plots of **Fig. 9d**.

The experiment was performed three times (**a**) with similar results.

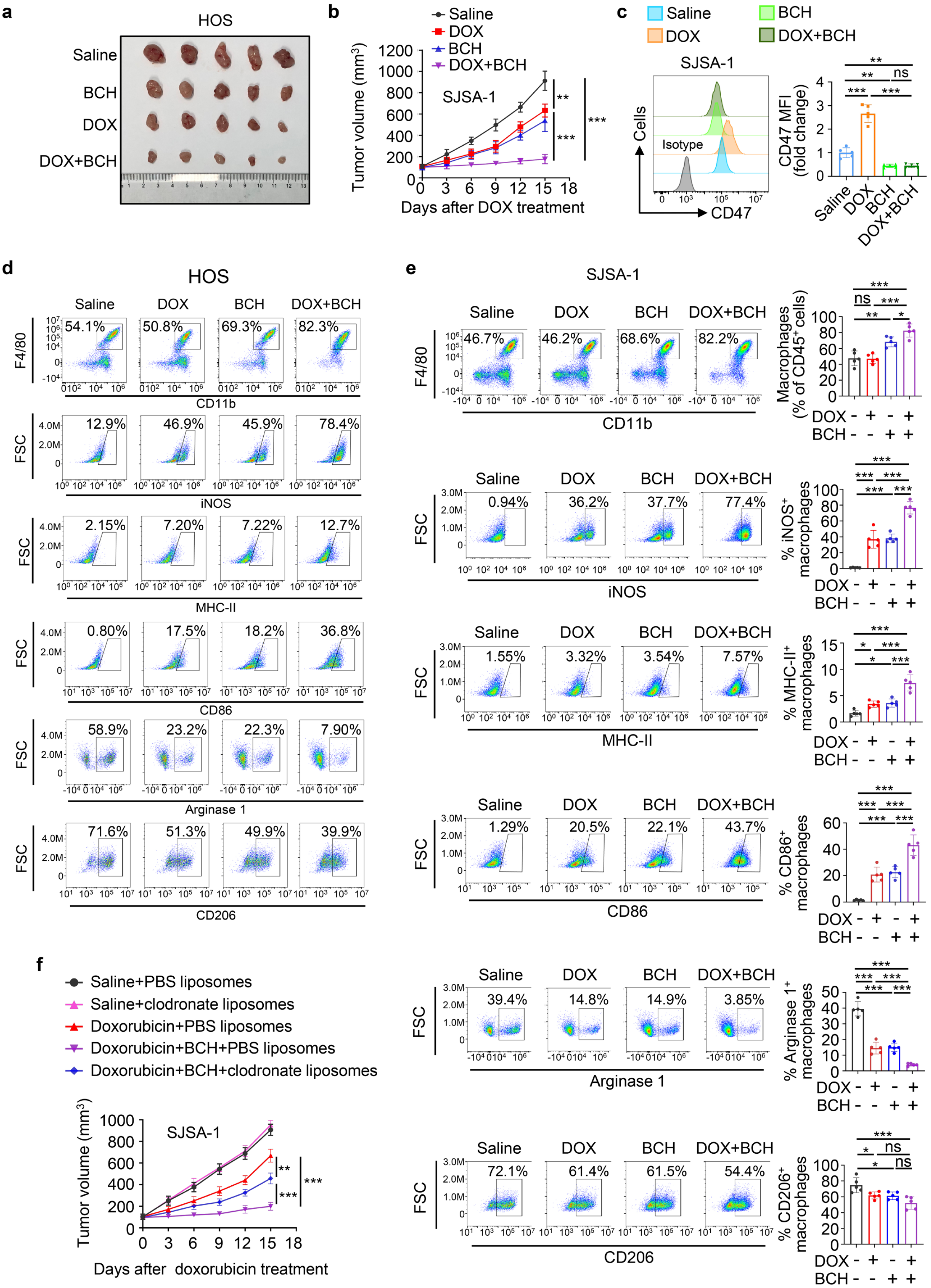
a
HOS
Saline
BCH
DOX
DOX+BCH
b
Saline
DOX
BCH
DOX+BCH
SJSA-1
Tumor volume (mm³)
Days after DOX treatment
c
Saline
DOX
BCH
DOX+BCH
SJSA-1
Isotype
Cells
CD47
CD47 MFI (fold change)
d
HOS
F4/80
CD11b
FSC
iNOS
MHC-II
CD86
Arginase 1
CD206
e
SJSA-1
Macrophages (% of CD45⁺ cells)
% iNOS⁺ macrophages
% MHC-II⁺ macrophages
% CD86⁺ macrophages
% Arginase 1⁺ macrophages
% CD206⁺ macrophages
f
Saline+PBS liposomes
Saline+clodronate liposomes
Doxorubicin+PBS liposomes
Doxorubicin+BCH+PBS liposomes
Doxorubicin+BCH+clodronate liposomes
Days after doxorubicin treatment

g

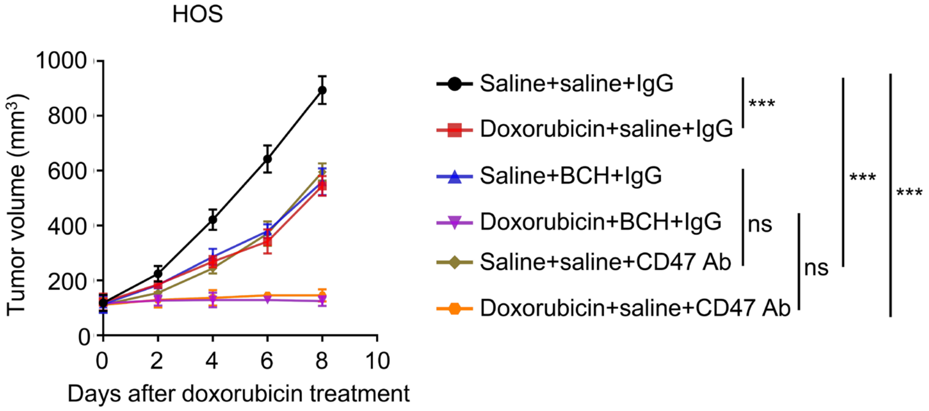


**Supplementary Figure 17, related to Fig. 10. BCH sensitizes osteosarcoma to doxorubicin treatment.**

**a** Representative images of HOS tumors treated as described in **Fig. 10a-d** on day 22.

**b**, **c**, **e** SJSA-1 tumor-bearing mice were treated with doxorubicin (DOX) along with BCH, after tumors grew for 14 days. **b** Tumor growth in mice (*n*=5 mice per group). Tumor volume was measured at the indicated time points. **c** Flow cytometric analysis of CD47 protein expression in SJSA-1 cells from tumors on day 22 (left; *n*=5 mice per group). SJSA-1 cells were additionally labeled with GFP and GFP positive events that represent SJSA-1 cells were gated for CD47 analysis. The anti-CD47 median fluorescence intensity (MFI) was determined (right). **e** Flow cytometric analysis of MHC-II, iNOS, CD86, Arginase 1, and CD206 expression in $CD45^+$ $CD11b^+$ $F4/80^+$ macrophages in SJSA-1 tumors on day 22 (*n*=5 mice per group).

**d** Representative flow plots of **Fig. 10d**.

**f** Tumor growth of SJSA-1 cells in mice treated as indicated (*n*=5 mice per group). Tumor volume was measured at the indicated time points.

**g** HOS tumor-bearing mice were treated with doxorubicin along with BCH or CD47 antibody after tumors grew for 14 days. Tumor growth in mice was monitored (*n*=5 mice per group). Tumor volume was measured at the indicated time points.

Data are shown as the mean ± SD. ns, not significant. **P* < 0.05, ***P* < 0.01, ****P* < 0.001, two-way ANOVA (**b**, **f**, **g**) or one-way ANOVA (**c**, **e**). The experiment was performed three times (**a-c**, **e**, **g**) or twice (**f**) with similar results. See Source Data file for the exact *P*-values. Source data are provided as a Source Data file.

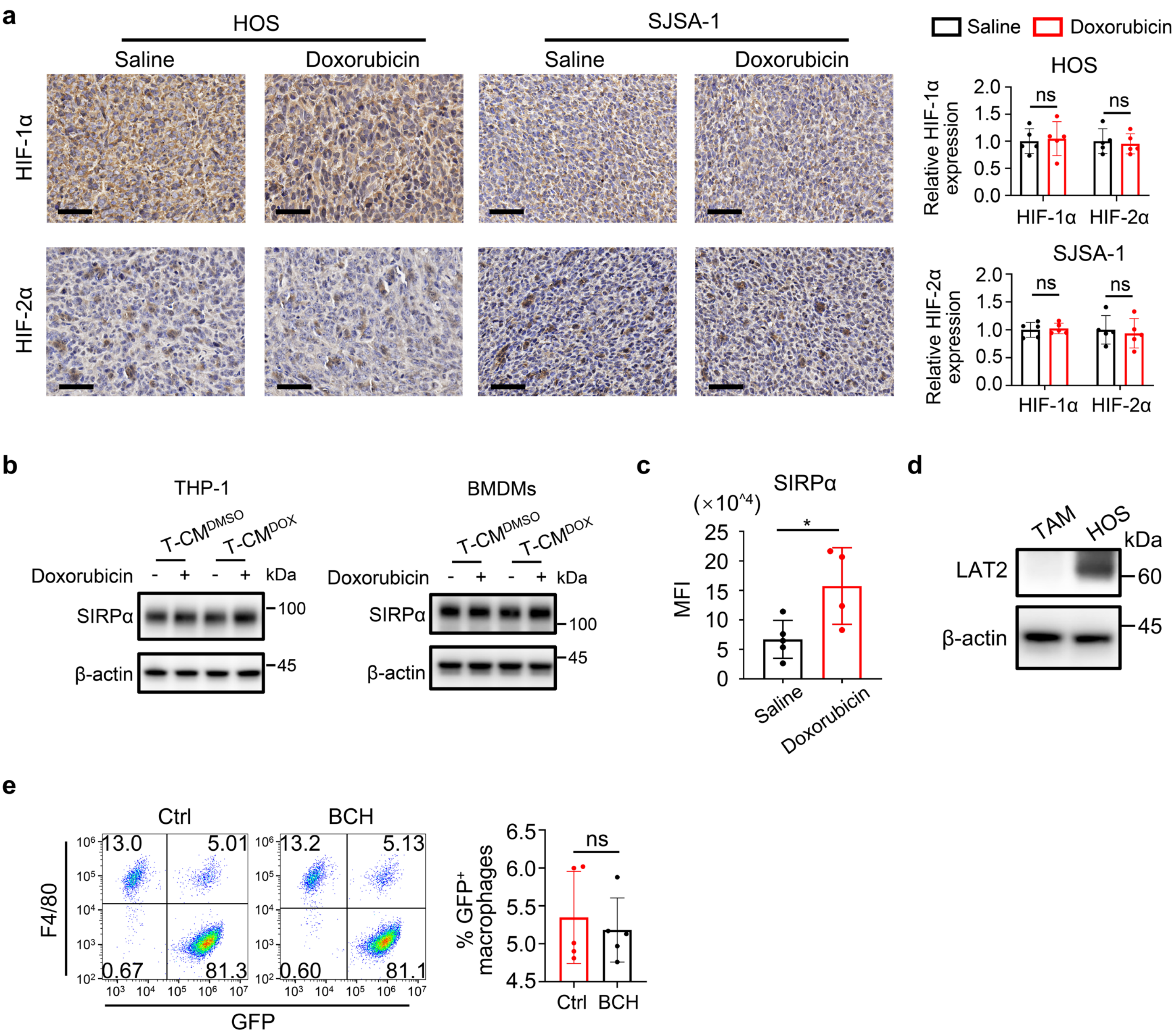


**Supplementary Figure 18, related to Discussion.**

**a** Immunohistochemical staining with antibodies as indicated in serial sections of HOS or SJSA-1 tumors treated with saline or doxorubicin on day 22 or day 29, respectively. Scale bars, 50 μm. Representative images are shown on the left. Statistical analyses of quantification results are shown on the right (*n*=5 mice per group).

**b** Western blot analysis of THP-1 derived macrophages (left) or bone marrow-derived macrophages (BMDMs; right) treated with or without doxorubicin (1 μM), in the presence of DMSO- or doxorubicin-treated HOS conditioned medium (T-CM$^{DMSO}$ or T-CM$^{DOX}$), for 24 h.

**c** Mice were treated with saline or doxorubicin every 2 days after HOS tumors grew for 14 days (*n*=4 mice per group). Flow cytometric analysis of SIRPα expression in $CD45^+$ $CD11b^+$ $F4/80^+$ macrophages in tumors on day 22.

**d** $CD45^+$ $CD11b^+$ $F4/80^+$ tumor associated macrophages (TAMs) and $GFP^+$ HOS cells were sorted by fluorescence-activated cell sorting after tumor grew for 20 days. Western blot analysis of LAT2 expression in TAMs and HOS cells.

**e** Flow cytometry-based in vitro macrophage phagocytosis assay (*n*=5 independent experiments). Bone marrow-derived macrophages (BMDMs) were pre-treated with BCH (25 mM; Sigma-Aldrich) for 24 h before co-culture with HOS cells. Macrophages were defined as $F4/80^+$ (labeled with PE) events, and tumor cells as $GFP^+$ events. $F4/80^+$, $GFP^+$ events represented macrophages that had phagocytosed tumor cells.

Data are shown as the mean ± SD. ns, not significant. *$P < 0.05$, unpaired two-tailed Student t test (**a**, **c**, **e**). The experiment was performed three times (**a**-**d**) with similar results. See Source Data file for the exact *P*-values. Source data are provided as a Source Data file.

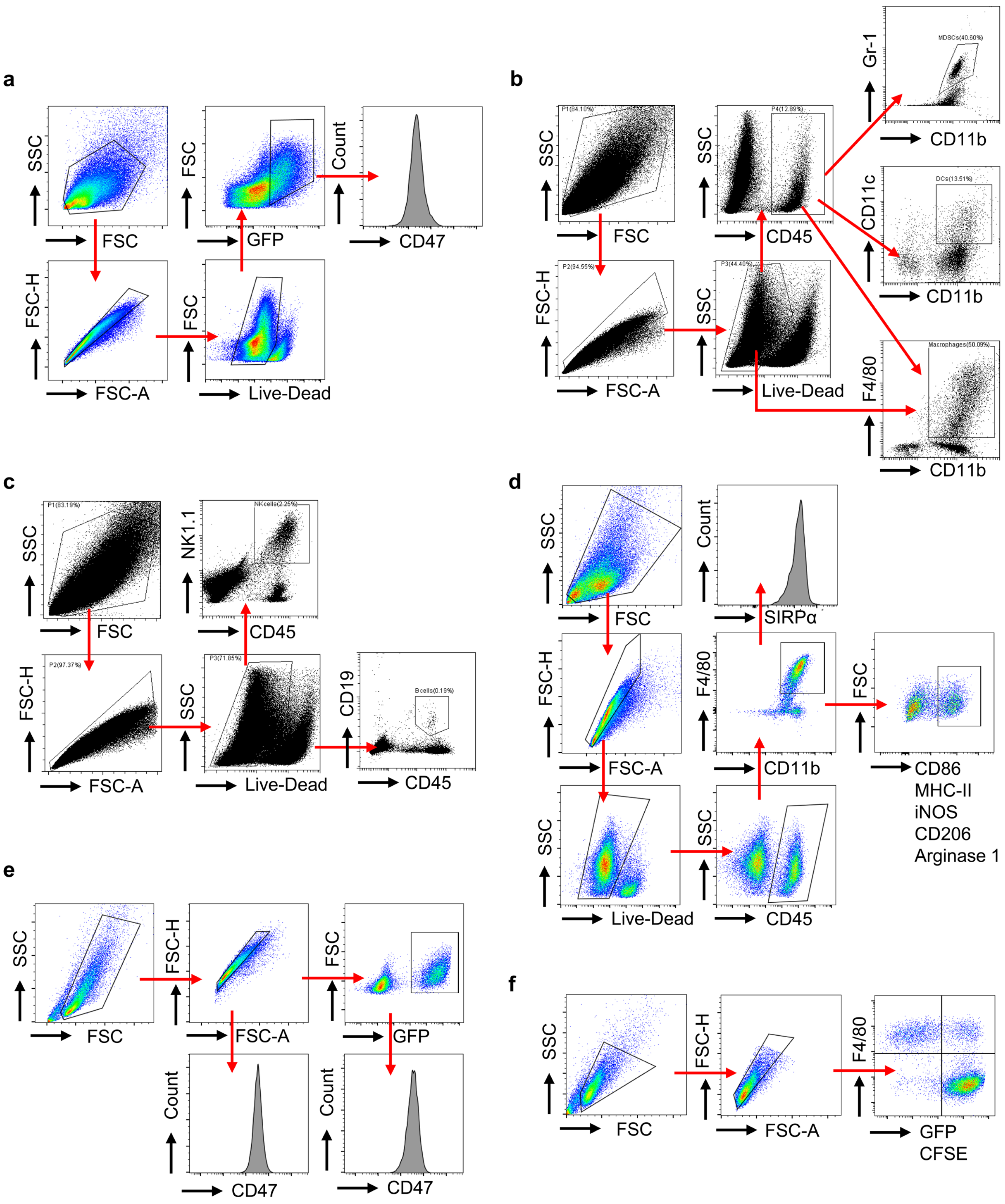


**Supplementary Figure 19. Gating strategies of flow cytometry analyses.**

**a** Gating strategies of Fig. 1c and Supplementary Figs. 15a, 17c, 18d.

**b** Gating strategies of Supplementary Figs. 2i-k, 2q, 13d.

**c** Gating strategies of Supplementary Fig. 2l, m.

**d** Gating strategies of Figs. 2a-e, 9d, 10d and Supplementary Figs. 15c, 16b, 17d, 17e, 18c.

**e** Gating strategies of Fig. 3b and Supplementary Fig. 3a, 3b.

**f** Gating strategies of Figs. 3d, 3e, 4c, 4g, 7f, 8b, 4e, 6d, 6e, 9g, 12b, 13c-f, 13h, 14b, 14d, 18e.

## Supplementary tables

**Supplementary Table 1. Canonical markers for the seven cell clusters in osteosarcoma lesions.**

| **Cell cluster** | **Marker genes**[1] |
|---|---|
| Osteoblastic cells | *RUNX2*, *COL1A1*, *CDH11*, *IBSP* |
| Osteoclasts | *ACP5*, *CTSK*, *MMP9* |
| T cells | *CD3D* |
| Macrophages | *CD14*, *CD68* |
| Mesenchymal stem cells | *MME*, *THY1*, *CXCL12* |
| Endothelial cells | *PECAM1*, *VWF* |

**Supplementary Table 2. Characteristics of osteosarcoma patients included in this study.**

| Characteristic | | Overall |
|---|---|---|
| Age at diagnosis (years) | ≥20 | 61 |
| | <20 | 123 |
| Sex | Male | 91 |
| | Femal | 93 |
| Tumor location | Femur | 117 |
| | Tibia | 35 |
| | Humerus | 14 |
| | Ulna | 5 |
| | Fibula | 3 |
| | Radius | 4 |
| | Others | 6 |
| Metastasis | Present | 98 |
| | Absent | 86 |
| Chemotherapy regimen | MAP | 171 |
| | MAPI | 13 |
| Histological subtype | Conventional osteosarcoma | 131 |
| | -Osteoblastic | 103 |
| | -Chondroblastic | 25 |
| | -Fibroblastic | 3 |
| | Telangiectatic | 11 |
| | Parosteal | 12 |
| | Others | 3 |
| | Unknown | 27 |

**Supplementary Table 3. Information of antibodies used in this study. See Reporting Summary for information on antibody validation.**

| **Antibodies used for western blot** | | | | |
|---|---|---|---|---|
| **Antibodies** | **Supplier** | **Catalog number** | **Clone name** | **Dilution ratio** |
| CD47 | Proteintech | 66304-1-Ig | 1E1D8 | 1:1000 |
| Phospho-p70 S6 Kinase (Thr389) | CST | 9234 | 108D2 | 1:1000 |
| p70 S6 Kinase | CST | 9202 | NA | 1:1000 |
| LAT2 | HUABIO | ER62494 | NA | 1:1000 |
| c-MYC | Abcam | ab32072 | Y69 | 1:1000 |
| CD47 | Abcam | ab175388 | NA | 1:1000 |
| SIRPα | Abcam | Ab191419 | EPR16264 | 1:1000 |
| GAPDH | Abcam | ab8245 | 6C5 | 1:5000 |
| β-actin | Abcam | ab6276 | AC-15 | 1:10000 |
| HRP conjugated goat anti-mouse IgG (H+L) | BOSTER | BA1051 | NA | 1:5000 |
| HRP conjugated goat anti-rabbit IgG (H+L) | BOSTER | BA1055 | NA | 1:5000 |
| **Antibodies used for flow cytometry** | | | | |
| **Antibodies** | **Supplier** | **Catalog number** | **Clone name** | **Dilution ratio** |
| Brilliant Violet 605™ anti-mouse CD45 | BioLegend | 103139 | 30-F11 | 1:200 |
| APC/Fire™ 750 anti-mouse CD11c | BioLegend | 117351 | N418 | 1:200 |
| APC anti-mouse CD206 (MMR) | BioLegend | 141708 | C068C2 | 1:200 |
| PE anti-mouse F4/80 | BioLegend | 157304 | QA17A29 | 1:200 |
| PerCP anti-mouse/human CD11b | BioLegend | 101230 | M1/70 | 1:200 |
| FITC anti-mouse CD86 | BioLegend | 105006 | GL-1 | 1:200 |
| APC anti-mouse CD19 | BioLegend | 152409 | 1D3/CD19 | 1:200 |
| FITC anti-mouse NK-1.1 | BioLegend | 156507 | S17016D | 1:200 |
| Alexa Fluor® 700 anti-mouse Ly-6G/Ly-6C | BioLegend | 108422 | RB6-8C5 | 1:200 |
| APC anti-human CD47 | BioLegend | 323123 | CC2C6 | 1:200 |
| eFluor™ 450 anti-mouse I-A/I-E | eBioscience | 48-5321-82 | M5/114.15.2 | 1:200 |
| APC-eFluor 780 anti-mouse iNOS | eBioscience | 47-5920-82 | CXNFT | 1:200 |
| PE-Cyanine7 anti-mouse Arginase 1 | eBioscience | 25-3697-82 | A1exF5 | 1:200 |
| APC anti-mouse SIRPα | BioLegend | 144014 | P84 | 1:200 |
| APC Rat IgG1, κ isotype Ctrl | BioLegend | 400412 | RTK2071 | 1:200 |
| APC Mouse IgG1, κ isotype Ctrl | Biolegend | 400120 | MOPC-21 | 1:200 |
| **Antibodies used for immunofluorescence** | | | | |
| **Antibodies** | **Supplier** | **Catalog number** | **Clone name** | **Dilution ratio** |

| | | | | |
|---|---|---|---|---|
| CD47 | Abcam | ab175388 | NA | 1:1000 |
| PE anti-mouse F4/80 | BioLegend | 157304 | QA17A29 | 1:200 |
| Alexa Fluor 555-labeled donkey anti-rabbit IgG(H+L) | Beyotime | A0453 | NA | 1:500 |
| **Antibodies used for immunohistochemistry** | | | | |
| **Antibodies** | **Supplier** | **Catalog number** | **Clone name** | **Dilution ratio** |
| CD47 | Abcam | ab218810 | EPR21794 | 1:2000 |
| IL-18 | Abcam | ab243091 | EPR19954-188 | 1:1000 |
| IL-18 | Abcam | ab223293 | EPR22249-212 | 1:1000 |
| F4/80 | CST | 70076 | D2S9R | 1:500 |
| CD86 | CST | 19589 | E5W6H | 1:250 |
| CD14 | Proteintech | 17000-1-AP | 2C1D9 | 1:1000 |
| CD206 | Proteintech | 60143-1-Ig | 2A6A10 | 1:20000 |
| iNOS | Abcam | ab283655 | RM1017 | 1:2000 |
| LAT2 | OriGene | TA500503 | OTI5A9 | 1:50 |
| HIF-1α | Abcam | ab51608 | EP1215Y | 1:200 |
| HIF-2α | Abcam | ab109616 | NA | 1:800 |
| HRP conjugated polyclonal anti-rabbit IgG | BOSTER | SV0002 | NA | 1:1 |
| HRP conjugated polyclonal anti-mouse IgG | BOSTER | SV0001 | NA | 1:1 |
| **In vitro CD47 blockade** | | | | |
| **Antibodies** | **Supplier** | **Catalog number** | **Clone name** | **Concentration** |
| CD47 | Bio X cell | BP0283 | MIAP410 | 20 ug/ml |
| IgG1 | Bio X cell | BE0083 | MOPC-21 | 20 ug/ml |
| **In vivo CD47 blockade** | | | | |
| **Antibodies** | **Supplier** | **Catalog number** | **Clone name** | **Concentration** |
| CD47 | Bio X cell | BP0283 | MIAP410 | 10 mg/kg |
| IgG1 | Bio X cell | BE0083 | MOPC-21 | 10 mg/kg |

CST, Cell Signaling Technology.

**Supplementary Table 4. Amino acids used in this study. *All amino acids were purchased from Sigma Aldrich.**

| Amino acid | Item number |
|---|---|
| L-Arginine monohydrochloride | A6969 |
| L-Cystine dihydrochloride | C6727 |
| L-Glutamine | G8540 |
| Glycine | G8790 |
| L-Histidine monohydrochloride monohydrate | H5659 |
| L-Isoleucine | I7403 |
| L-Leucine | L8912 |
| L-Lysine monohydrochloride | L8662 |
| L-Methionine | M5308 |
| L-Phenylalanine | P5482 |
| L-Serine | S4311 |
| L-Threonine | T8441 |
| L-Tryptophan | T8941 |
| L-Tyrosine disodium salt hydrate | T1145 |
| L-Valine | V0513 |

**Supplementary Table 5. Primers used in this study.**

| Gene | Species | Orientation | Sequence (5' to 3') | Source |
|---|---|---|---|---|
| *Itgam* (*Cd11b*) | Mouse | Forward | CCATGACCTTCCAAGAGAATGC | PrimerBank ID: 132626288c1 |
| *Itgam* | Mouse | Reverse | ACCGGCTTGTGCTGTAGTC | |
| *F4/80* | Mouse | Forward | CTGCACCTGTAAACGAGGCTT | PrimerBank ID: 183583543c1 |
| *F4/80* | Mouse | Reverse | GCAGACTGAGTTAGGACCACAA | |
| *Cd80* | Mouse | Forward | TCAGTTGATGCAGGATACACCA | PrimerBank ID: 111038144c1 |
| *Cd80* | Mouse | Reverse | AAAGACGAATCAGCAGCACAA | |
| *Cd86* | Mouse | Forward | TCAATGGGACTGCATATCTGCC | PrimerBank ID: 161484598c1 |
| *Cd86* | Mouse | Reverse | GCCAAAATACTACCAGCTCACT | |
| *Cd163* | Mouse | Forward | GGTGGACACAGAATGGTTCTTC | PrimerBank ID: 281371422c1 |
| *Cd163* | Mouse | Reverse | CCAGGAGCGTTAGTGACAGC | |
| *Mrc1* (*Cd206*) | Mouse | Forward | CTCTGTTCAGCTATTGGACGC | PrimerBank ID: 224967061c1 |
| *Mrc1* | Mouse | Reverse | TGGCACTCCCAAACATAATTTGA | |
| *Nos2* | Mouse | Forward | GTTCTCAGCCCAACAATACAAGA | PrimerBank ID: 6754872a1 |
| *Nos2* | Mouse | Reverse | GTGGACGGGTCGATGTCAC | |
| *Arg1* | Mouse | Forward | CTCCAAGCCAAAGTCCTTAGAG | PrimerBank ID: 7106255a1 |
| *Arg1* | Mouse | Reverse | AGGAGCTGTCATTAGGGACATC | |
| *Actb* | Mouse | Forward | CATTGCTGACAGGATGCAGAAGG | #MP200232 |
| *Actb* | Mouse | Reverse | TGCTGGAAGGTGGACAGTGAGG | |
| *CD47* | Human | Forward | AGAAGGTGAAACGATCATCGAGC | PrimerBank ID: 68223312c1 |
| *CD47* | Human | Reverse | CTCATCCATACCACCGGATCT | |
| *CD80* | Human | Forward | AAACTCGCATCTACTGGCAAA | PrimerBank ID: 113722122c1 |
| *CD80* | Human | Reverse | GGTTCTTGTACTCGGGCCATA | |
| *CD86* | Human | Forward | CTGCTCATCTATACACGGTTACC | PrimerBank ID: 332634933c1 |
| *CD86* | Human | Reverse | GGAAACGTCGTACAGTTCTGTG | |
| *CD163* | Human | Forward | TTTGTCAACTTGAGTCCCTTCAC | PrimerBank ID: 344179109c1 |
| *CD163* | Human | Reverse | TCCCGCTACACTTGTTTTCAC | |
| *MRC1* (*CD206*) | Human | Forward | TCCGGGTGCTGTTCTCCTA | PrimerBank ID: 145312260c1 |
| *MRC1* | Human | Reverse | CCAGTCTGTTTTTGATGGCACT | |
| *IL18R1* | Human | Forward | AAGAACGCCGAGTTTGAAGAT | PrimerBank ID: 27477086c2 |
| *IL18R1* | Human | Reverse | GAGCAGTTGAGCCTTACGTTT | |
| *SLC7A5* (*LAT1*) | Human | Forward | CCGTGAACTGCTACAGCGT | PrimerBank ID: 71979931c1 |
| *SLC7A5* | Human | Reverse | CTTCCCGATCTGGACGAAGC | |
| *SLC7A8* (*LAT2*) | Human | Forward | AGGCTGGAACTTTCTGAATTACG | PrimerBank ID: 33286427c1 |
| *SLC7A8* | Human | Reverse | ACATAAGCGACATTGGCAAAGA | |
| *SLC43A1* (*LAT3*) | Human | Forward | GGACGTGGAAGCTCTGTCTC | PrimerBank ID: 311771735c1 |
| *SLC43A1* | Human | Reverse | GCAGCGTGAGTGAAGTGAAC | |
| *SLC43A2* (*LAT4*) | Human | Forward | AGTCAGAGGGCTTTTACTCCTAC | PrimerBank ID: 22748750c1 |
| *SLC43A2* | Human | Reverse | GTCCATGACGATACCCAGGG | |
| *SLC3A2* (*4F2HC*) | Human | Forward | TGAATGAGTTAGAGCCCGAGA | PrimerBank ID: 312032447c1 |
| *SLC3A2* | Human | Reverse | GTCTTCCGCCACCTTGATCTT | |

| | | | | |
|---|---|---|---|---|
| *ACTB* | Human | Forward | CACCATTGGCAATGAGCGGTTC | #HP204660 |
| *ACTB* | Human | Reverse | AGGTCTTTGCGGATGTCCACGT | |

## Supplementary Reference

1. Zhou Y*, et al.* Single-cell RNA landscape of intratumoral heterogeneity and immunosuppressive microenvironment in advanced osteosarcoma. *Nature communications* **11**, 6322 (2020).
