# Supplementary material for: Metabolic control of CD47 expression through LAT2-mediated amino acid uptake promotes tumor immune evasion
Source: Nat Commun. 2022 Oct 23;13:6308. doi: 10.1038/s41467-022-34064-4 (PMC9588779; doi:10.1038/s41467-022-34064-4)
Supplement: Supplementary file 3 — Description of Additional Supplementary Files [file 41467_2022_34064_MOESM3_ESM.pdf]

Description of Additional Supplementary Files

Title: Supplementary Data 1

Description: The UHPLC-MS/MS based quantification of amino acids in shCtrl or shLAT2 HOS cells stimulated with PBS or IL-18 (40 ng/ml) for 24 h.

Title: Supplementary Data 2

Description: The UHPLC-MS/MS based quantification of leucine in shCtrl or shLAT2 HOS cells stimulated with PBS or IL-18 (40 ng/ml) for 24 h.
