# Supplementary material for: Metabolic control of CD47 expression through LAT2-mediated amino acid uptake promotes tumor immune evasion
Source: Nat Commun. 2022 Oct 23;13:6308. doi: 10.1038/s41467-022-34064-4 (PMC9588779; doi:10.1038/s41467-022-34064-4)
Supplement: Supplementary file 6 — Reporting Summary [file 41467_2022_34064_MOESM6_ESM.pdf]

nature portfolio

Corresponding author(s): Zhaoming Ye

Last updated by author(s): Oct 5, 2022

Reporting Summary

Nature Portfolio wishes to improve the reproducibility of the work that we publish. This form provides structure for consistency and transparency in reporting. For further information on Nature Portfolio policies, see our Editorial Policies and the Editorial Policy Checklist.

Statistics

For all statistical analyses, confirm that the following items are present in the figure legend, table legend, main text, or Methods section.

n/a	Confirmed	
☐	☒	The exact sample size (n) for each experimental group/condition, given as a discrete number and unit of measurement
☐	☒	A statement on whether measurements were taken from distinct samples or whether the same sample was measured repeatedly
☐	☒	The statistical test(s) used AND whether they are one- or two-sided<br>*Only common tests should be described solely by name; describe more complex techniques in the Methods section.*
☒	☐	A description of all covariates tested
☐	☒	A description of any assumptions or corrections, such as tests of normality and adjustment for multiple comparisons
☐	☒	A full description of the statistical parameters including central tendency (e.g. means) or other basic estimates (e.g. regression coefficient) AND variation (e.g. standard deviation) or associated estimates of uncertainty (e.g. confidence intervals)
☐	☒	For null hypothesis testing, the test statistic (e.g. F, t, r) with confidence intervals, effect sizes, degrees of freedom and P value noted<br>*Give P values as exact values whenever suitable.*
☒	☐	For Bayesian analysis, information on the choice of priors and Markov chain Monte Carlo settings
☒	☐	For hierarchical and complex designs, identification of the appropriate level for tests and full reporting of outcomes
☐	☒	Estimates of effect sizes (e.g. Cohen's d, Pearson's r), indicating how they were calculated

Our web collection on statistics for biologists contains articles on many of the points above.

Software and code

Policy information about availability of computer code

Data collection	CytExpert (version 2.4), Leica Application Suite X (version 3.7.4), Quantity One (version 4.6.9), Agilent MassHunter Work Station (version B.08.00), Summit (version 6.3.1), SoftMax Pro (version 7.1), ImageQuant 800 Control software (version 2.0.0), Real-Time PCR software (version 2.4)
Data analysis	FlowJo (version 10.4), Graphpad Prism (version 7.0.0), Medcalc (version 19.1.2), GSEA software (version 4.0.3), R Seurat package (version 3.2.3), X-tile (version 3.6.1), CIBERSORT, ImageJ (version 1.8.0), IBM SPSS Statistics 23

For manuscripts utilizing custom algorithms or software that are central to the research but not yet described in published literature, software must be made available to editors and reviewers. We strongly encourage code deposition in a community repository (e.g. GitHub). See the Nature Portfolio guidelines for submitting code & software for further information.

Data

Policy information about availability of data

All manuscripts must include a data availability statement. This statement should provide the following information, where applicable:

- Accession codes, unique identifiers, or web links for publicly available datasets
- A description of any restrictions on data availability
- For clinical datasets or third party data, please ensure that the statement adheres to our policy

The publicly available data used in Fig. 6a, b and Supplementary Fig. 6a are available in the GEO database with accession number GSE152048 (https://www.ncbi.nlm.nih.gov/geo/query/acc.cgi?acc=GSE152048). The publicly available data used in Fig. 6c, d and Supplementary Figs. 2p, 7a are generated by the TARGET (https://ocg.cancer.gov/programs/target) initiative, phs000468, with additional data available at https://portal.gdc.cancer.gov/projects. The publicly available data used in Fig. 6e and Supplementary Fig. 4a are available in the GEO database with accession number GSE21257 (https://www.ncbi.nlm.nih.gov/geo/

query/acc.cgi?acc=GSE21257), GSE30699 (https://www.ncbi.nlm.nih.gov/geo/query/acc.cgi?acc=GSE30699) and GSE33382 (https://www.ncbi.nlm.nih.gov/geo/query/acc.cgi?acc=GSE33382). The publicly available data used in Supplementary Fig. 2n, o are available in the GEO database with accession number GSE30699. The remaining data are available within the Article, Supplementary Information or Source Data file. Source data are provided with this paper.

Field-specific reporting

Please select the one below that is the best fit for your research. If you are not sure, read the appropriate sections before making your selection.

- [x] Life sciences
- [ ] Behavioural & social sciences
- [ ] Ecological, evolutionary & environmental sciences

For a reference copy of the document with all sections, see nature.com/documents/nr-reporting-summary-flat.pdf

Life sciences study design

All studies must disclose on these points even when the disclosure is negative.

Sample size	Sample-size calculations were not done. For in vivo and in vitro studies, sample sizes were determined based on common practices of the fields (e.g. PMID: 26512116, PMID: 22451913, PMID: 26322579, PMID: 32198351) and the magnitude and consistency of measurable differences between groups. n = 3-6 samples per group are sufficient to detect meaningful biological differences (precise samples size for each experiment is indicated in the figure legends). All experiments were performed with good reproducibility.
Data exclusions	No data were excluded from the analyses.
Replication	All experiments were performed at least 3 times. All experimental findings were reproduced.
Randomization	For in vivo experiments, age- and sex-matched mice were randomized based on tumour burden before they were assigned into different treatment groups. For in vitro experiments, all cells in each experiment were from the same pool of parental cells and were randomized into different treatment groups.
Blinding	The investigators were blinded to group allocation during data collection or analysis.

Reporting for specific materials, systems and methods

We require information from authors about some types of materials, experimental systems and methods used in many studies. Here, indicate whether each material, system or method listed is relevant to your study. If you are not sure if a list item applies to your research, read the appropriate section before selecting a response.

Materials & experimental systems

n/a	Involved in the study	
☐	☒	Antibodies
☐	☒	Eukaryotic cell lines
☒	☐	Palaeontology and archaeology
☐	☒	Animals and other organisms
☐	☒	Human research participants
☒	☐	Clinical data
☒	☐	Dual use research of concern

Methods

n/a	Involved in the study	
☒	☐	ChIP-seq
☐	☒	Flow cytometry
☒	☐	MRI-based neuroimaging

Antibodies

Antibodies used

The following primary antibodies were used for western blot. They are listed as antigen first, followed by supplier, catalog number, clone name and dilution ratio.
1)CD47, Proteictech, 66304-1-Ig, 1E1D8, 1:1000
2) Phospho-p70 S6 Kinase (Thr389), Cell Signaling Technology, 9234, 108D2, 1:1000
3) p70 S6 Kinase, Cell Signaling Technology, 9202, NA, 1:1000
4) LAT2, HUABIO, ER62494, NA, 1:1000
5) c-MYC, Abcam, ab32072, Y69, 1:1000
6) CD47, Abcam, ab175388, NA, 1:1000
7) SIRPα, Abcam, ab191419, EPR16264, 1:1000
8) GAPDH, Abcam, ab8245, 6C5, 1:5000
9) β-actin, Abcam, ab6276, AC-15, 1:10000
10) HRP conjugated goat anti-mouse IgG (H+L), BOSTER, BA1051, NA, 1:5000
11) HRP conjugated goat anti-rabbit IgG (H+L), BOSTER , BA1055, NA, 1:5000

The following primary antibodies were used for flow cytometry. They are listed as antigen first, followed by supplier, catalog number,

clone name and dilution ratio.
1) Brilliant Violet 605™ anti-mouse CD45, BioLegend, 103139, 30-F11, 1:200
2) APC/Fire™ 750 anti-mouse CD11c, BioLegend, 117351, N418, 1:200
3) APC anti-mouse CD206 (MMR), BioLegend, 141708, C068C2, 1:200
4) PE anti-mouse F4/80, BioLegend 157304, QA17A29, 1:200
5) PerCP anti-mouse/human CD11b, BioLegend, 101230, M1/70, 1:200
6) FITC anti-mouse CD86, BioLegend, 105006, GL-1, 1:200
7) APC anti-mouse CD19, BioLegend, 152409, 1D3/CD19, 1:200
8) FITC anti-mouse NK-1.1, BioLegend, 156507, S17016D, 1:200
9) Alexa Fluor® 700 anti-mouse Ly-6G/Ly-6C, BioLegend, 108422, RB6-8C5, 1:200
10) APC anti-human CD47, BioLegend, 323123, CC2C6, 1:200
11) eFluor™ 450 anti-mouse I-A/I-E, eBioscience, 48-5321-82, M5/114.15.2, 1:200
12) APC-eFluor 780 anti-mouse iNOS, eBioscience, 47-5920-82, CXNFT, 1:200
13) PE-Cyanine7 anti-mouse Arginase 1, eBioscience, 25-3697-82, A1exF5, 1:200
14) APC anti-mouse SIRPα, BioLegend, 144014, P84, 1:200
15) APC Rat IgG1, κ isotype Ctrl, BioLegend, 400412, RTK2071, 1:200
16) APC Mouse IgG1, κ isotype Ctrl, Biolegend, 400120, MOPC-21, 1:200

The following primary antibodies were used for immunofluorescence. They are listed as antigen first, followed by supplier, catalog number, clone name and dilution ratio.
1) CD47, Abcam, ab175388, NA, 1:1000
2) PE anti-mouse F4/80, BioLegend, 157304, QA17A29, 1:200
3) Alexa Fluor 555-labeled donkey anti-rabbit IgG(H+L), Beyotime, A0453, NA, 1:500

The following primary antibodies were used for immunohistochemistry. They are listed as antigen first, followed by supplier, catalog number, clone name and dilution ratio.
1) CD47, Abcam, ab218810, EPR21794, 1:2000
2) IL-18, Abcam, ab243091, EPR19954-188, 1:1000
3) IL-18, Abcam, ab223293, EPR22249-212, 1:1000
4) F4/80, Cell Signaling Technology, 70076, D2S9R, 1:500
5) CD86, Cell Signaling Technology, 19589, E5W6H, 1:250
6) CD14, Proteintech, 17000-1-AP, 2C1D9, 1:1000
7) CD206, Proteintech, 60143-1-Ig, 2A6A10, 1:20000
8) iNOS, Abcam, ab283655, RM1017, 1:2000
9) LAT2, OriGene, TA500503, OTI5A9, 1:50
10) HIF-1α, Abcam, ab51608, EP1215Y, 1:200
11) HIF-2α, Abcam, ab109616, NA, 1:800
12) HRP conjugated polyclonal anti-rabbit IgG, BOSTER, SV0002, NA, 1:1
13) HRP conjugated polyclonal anti-mouse IgG, BOSTER, SV0001, NA, 1:1

The following primary antibodies were used for in vitro CD47 blockade. They are listed as antigen first, followed by supplier, catalog number, clone name and dilution ratio.
1) CD47, Bio X cell, BP0283, MIAP410, 20 ug/ml
2) IgG1, Bio X cell, BE0083, MOPC-21, 20 ug/ml

The following primary antibodies were used for in vivo CD47 blockade. They are listed as antigen first, followed by supplier, catalog number, clone name and dilution ratio.
1) CD47, Bio X cell, BP0283, MIAP410, 10 mg/kg
2) IgG1, Bio X cell, BE0083, MOPC-21, 10 mg/kg

Validation

All antibodies were commercially available, and validated by manufacturers and/or citations. Manufacturer websites containing their validation data and/or citations, are listed below:
WB antibodies:
1) CD47 (Proteinch, cat.n. 66304-1-Ig): website (https://www.ptgcn.com/products/CD47-Antibody-66304-1-Ig.htm) and citations (PMID: 35381274 (human), etc).
2) Phospho-p70 S6 Kinase (Thr389) (Cell Signaling Technology, cat.n. 9234): website (https://www.cellsignal.cn/products/primary-antibodies/phospho-p70-s6-kinase-thr389-108d2-rabbit-mab/9234?site-search type=Products&N=4294956287&Ntt=9234&fromPage=plp) and citations (PMID: 36151083 (human), etc).
3) p70 S6 Kinase (Cell Signaling Technology, cat.n. 9202): website (https://www.cellsignal.cn/products/primary-antibodies/p70-s6-kinase-antibody/9202?site-search-type=Products&N=4294956287&Ntt=9202&fromPage=plp&_requestid=1994797) and citations (PMID: 36115844 (human), etc).
4) LAT2 (HUABIO, cat.n. ER62494): website (http://www.huabio.cn/product/LAT2-antibody-ER62494).
5) c-MYC (Abcam, cat.n. ab32072): website (https://www.abcam.cn/c-myc-antibody-y69-chip-grade-ab32072.html) and citations (PMID: 36068222 (human), etc).
6) CD47 (Abcam, cat.n. ab175388): website (https://www.abcam.cn/cd47-antibody-ab175388.html) and citations (PMID: 36096529 (human), etc).
7) SIRPα, Abcam, cat.n. ab191419): website (https://www.abcam.cn/sirp-alpha-antibody-epr16264-ab191419.html) and citations (PMID: 35127380 (human), PMID: 35585990 (mouse), etc).
8) GAPDH (Abcam, cat.n. ab8245): website (https://www.abcam.cn/gapdh-antibody-6c5-loading-control-ab8245.html) and citations

(PMID: 34706226 (human), etc).
9) β-actin (Abcam, cat.n. ab6276): website (https://www.abcam.cn/beta-actin-antibody-ac-15-ab6276.html) and citations (PMID: 33762331 (human), PMID: 31772164 (mouse), etc).
10) HRP conjugated goat anti-mouse IgG (H+L) (BOSTER, cat.n. BA1051): website (https://www.boster.com.cn/home/product/hrp-conjugated-affinipure-goat-anti-mouse-igg-h-l_ba1051.html) and citations (PMID: 21543155, etc).
11) HRP conjugated goat anti-rabbit IgG (H+L) (BOSTER , cat.n. BA1055): website (https://www.boster.com.cn/home/product/hrp-conjugated-affinipure-goat-anti-rabbit-igg-h-l_ba1055.html) and citations (PMID: 26332703, etc).

Flow cytometry antibodies
1) Brilliant Violet 605™ anti-mouse CD45 (BioLegend, cat.n. 103139): website (https://www.biolegend.com/en-gb/products/brilliant-violet-605-anti-mouse-cd45-antibody-8721).
2) APC/Fire™ 750 anti-mouse CD11c (BioLegend, cat.n. 117351): website (https://www.biolegend.com/en-gb/products/apc-fire-750-anti-mouse-cd11c-antibody-13050).
3) APC anti-mouse CD206 (MMR) (BioLegend, cat.n. 141708): website (https://www.biolegend.com/en-gb/products/apc-anti-mouse-cd206-mmr-antibody-7425).
4) PE anti-mouse F4/80 (BioLegend, cat.n. 157304): website (https://www.biolegend.com/en-gb/products/pe-anti-mouse-f4-80-recombinant-antibody-18755).
5) PerCP anti-mouse/human CD11b (BioLegend, cat.n. 101230): website (https://www.biolegend.com/en-gb/products/percp-anti-mouse-human-cd11b-antibody-4315).
6) FITC anti-mouse CD86 (BioLegend, cat.n. 105006): website (https://www.biolegend.com/en-gb/products/fitc-anti-mouse-cd86-antibody-254).
7) APC anti-mouse CD19 (BioLegend, cat.n. 152409): website (https://www.biolegend.com/en-gb/products/apc-anti-mouse-cd19-antibody-13680).
8) FITC anti-mouse NK-1.1 (BioLegend, cat.n. 156507): website (https://www.biolegend.com/en-gb/products/fitc-anti-mouse-nk-11-antibody-19869).
9) Alexa Fluor® 700 anti-mouse Ly-6G/Ly-6C (BioLegend, cat.n. 108422): website (https://www.biolegend.com/en-gb/products/alexa-fluor-700-anti-mouse-ly-6g-ly-6c-gr-1-antibody-3390).
10) APC anti-human CD47 (BioLegend, cat.n. 323123): website (https://www.biolegend.com/en-gb/products/apc-anti-human-cd47-antibody-14976).
11) eFluor™ 450 anti-mouse I-A/I-E (eBioscience, cat.n. 48-5321-82): website (https://www.thermofisher.cn/cn/zh/antibody/product/MHC-Class-II-I-A-I-E-Antibody-clone-M5-114-15-2-Monoclonal/48-5321-82).
12) APC-eFluor 780 anti-mouse iNOS (eBioscience, cat.n. 47-5920-82): website (https://www.thermofisher.cn/cn/zh/antibody/product/iNOS-Antibody-clone-CXNFT-Monoclonal/47-5920-82).
13) PE-Cyanine7 anti-mouse Arginase 1 (eBioscience, cat.n. 25-3697-82): website (https://www.thermofisher.cn/cn/zh/antibody/product/Arginase-1-Antibody-clone-A1exF5-Monoclonal/25-3697-82).
14) APC anti-mouse SIRPα (BioLegend, cat.n. 144014): website (https://www.biolegend.com/en-gb/products/apc-anti-mouse-cd172a-sirpalpha-antibody-10194).
15) APC Rat IgG1, κ isotype Ctrl (BioLegend, cat.n. 400412): website (https://www.biolegend.com/en-gb/products/apc-rat-igg1-kappa-isotype-ctrl-1826).
16) APC Mouse IgG1, κ isotype Ctrl (Biolegend, cat.n. 400120): website (https://www.biolegend.com/en-gb/products/apc-mouse-igg1-kappa-isotype-ctrl-1404).

Immunofluorescence antibodies:
1) CD47 (Abcam, cat.n. ab175388): website (https://www.abcam.cn/cd47-antibody-ab175388.html).
2) PE anti-mouse F4/80 (BioLegend, cat.n. 157304): website (https://www.biolegend.com/en-gb/products/pe-anti-mouse-f4-80-recombinant-antibody-18755).
3) Alexa Fluor 555-labeled donkey anti-rabbit IgG(H+L) (Beyotime, cat.n. A0453): website (https://www.beyotime.com/product/A0453.htm).

Immunohistochemistry antibodies:
1) CD47 (Abcam, cat.n. ab218810): website (https://www.abcam.cn/cd47-antibody-epr21794-ab218810.html).
2) IL-18 (Abcam, cat.n. ab243091): website (https://www.abcam.cn/il-18-antibody-epr19954-188-ab243091.html).
3) IL-18 (Abcam, cat.n. ab223293): website (https://www.abcam.cn/il-18-antibody-epr22249-212-ab223293.html).
4) F4/80 (Cell Signaling Technology, cat.n. 70076): website (https://www.cellsignal.cn/products/primary-antibodies/f4-80-d2s9r-xp-rabbit-mab/70076?site-search-type=Products&N=4294956287&Ntt=70076&fromPage=plp&_requestid=2022357).
5) CD86 (Cell Signaling Technology, cat.n. 19589): website (https://www.cellsignal.cn/products/primary-antibodies/cd86-e5w6h-rabbit-mab/19589?site-search-type=Products&N=4294956287&Ntt=19589&fromPage=plp&_requestid=2022860).
6) CD14 (Proteintech, cat.n. 17000-1-AP): website (https://www.ptgcn.com/products/CD14-Antibody-17000-1-AP.htm).
7) CD206 (Proteintech, cat.n. 60143-1-Ig): website (https://www.ptgcn.com/products/MRC1-Antibody-60143-1-Ig.htm).
8) iNOS (Abcam, act.n. ab283655): website (https://www.abcam.cn/inos-antibody-rm1017-ab283655.html).
9) LAT2 (OriGene, act.n. TA500503): website (https://www.origene.com.cn/catalog/antibodies/primary-antibodies/ta500503/lat2-slc7a8-mouse-monoclonal-antibody-clone-id-oti5a9).
10) HIF-1α (Abcam, cat.n. ab51608): website (https://www.abcam.cn/hif-1-alpha-antibody-ep1215y-ab51608.html).
11) HIF-2α (Abcam, cat.n. ab109616): website (https://www.abcam.cn/hif-2-alpha-antibody-ab109616.html).
12) HRP conjugated polyclonal anti-rabbit IgG (BOSTER, cat.n. SV0002): website (https://www.boster.com.cn/home/product/igg-hrp_sv0002.html).
13) HRP conjugated polyclonal anti-mouse IgG BOSTER, cat.n. SV0001): website (https://www.boster.com.cn/home/product/igg-hrp_sv0001.html).

In vitro and in vivo CD47 blockade antibodies:

	1) CD47 (Bio X cell, act.n. BP0283): webcite (https://bxcell.com/product/invivoplus-anti-mouse-human-rat-cd47-iap/) and citations (PMID: 22451913).<br>2) IgG1 (Bio X cell, act.n. BE0083): website (https://bxcell.com/product/mouse-igg1-isotype-control/) and citations (PMID: 23793061).

Eukaryotic cell lines

Policy information about cell lines

Cell line source(s)	The human osteosarcoma cell lines, U-2 OS, MG63, MNNG/HOS, the human osteoblast cell line, hFOB1.19, the human monocytic cell line, THP-1 and the human embryonic kidney cell line, 293T were obtained from Cell Collection of the Chinese Academy of Sciences. The human osteosarcoma cell line, 143B, was obtained from American Type Culture Collection (ATCC). The human osteosarcoma cell line, SJSA-1, was obtained from China Center for Type Culture Collection (CCTCC).
Authentication	U-2 OS, MG63, MNNG/HOS, hFOB1.19, and THP-1 cell lines were authenticated at Cell Collection of the Chinese Academy of Sciences through STR profiling. 143B cell line was authenticated at ATCC through STR profiling. The human osteosarcoma cell line, SJSA-1, was authenticated at CCTCC through STR profiling.
Mycoplasma contamination	All cell lines tested negative for mycoplasma contamination.
Commonly misidentified lines<br>(See ICLAC register)	No commonly misidentified cell lines are used in the study.

Animals and other organisms

Policy information about studies involving animals; ARRIVE guidelines recommended for reporting animal research

Laboratory animals	BALB/c nude mice: female, 5-week-old. BALB/c mice: female, 8-week-old. All mice were fed free access to water, housed in a 12 h light/dark cyle-, temperature- and humidity- controlled, pathogen-free facility. All mice were conducted adhering to the guidelines approved by the Ethics Committee of Second Affiliated Hospital, Zhejiang University School of Medicine (SAHZU).
Wild animals	No wild animals were included in this study.
Field-collected samples	No field-collected samples were used in this study.
Ethics oversight	All studies were conducted in accordance with the National Institute of Health Guide for the Care and Use of Laboratory Animals with approval of the Ethics Committee of SAHZU.

Note that full information on the approval of the study protocol must also be provided in the manuscript.

Human research participants

Policy information about studies involving human research participants

Population characteristics	Formalin-fixed, paraffin-embedded osteosarcoma pre- and post-chemotherapy tissue blocks were collected from the Second Affiliated Hospital, Zhejiang University School of Medicine. All of these human research participants have no age or gender preference: 91 males, 93 female; age from 9-61 years and 123 patients under 20 years, 61 patients equal to/over 20 years.
Recruitment	All patients were selected from the Second Affiliated Hospital, Zhejiang University School of Medicine. Most eligible patients agreed to participation. As a consequence, self-selection bias or other biases did not present in this study.
Ethics oversight	The collection and use of human samples were approved by the Ethics Committee of the Second Affiliated Hospital, Zhejiang University School of Medicine.

Note that full information on the approval of the study protocol must also be provided in the manuscript.

Flow Cytometry

Plots

Confirm that:

- [x] The axis labels state the marker and fluorochrome used (e.g. CD4-FITC).
- [x] The axis scales are clearly visible. Include numbers along axes only for bottom left plot of group (a 'group' is an analysis of identical markers).
- [x] All plots are contour plots with outliers or pseudocolor plots.
- [x] A numerical value for number of cells or percentage (with statistics) is provided.

Methodology

Sample preparation	For cultured cell lines, cells were harvested using cell scrapers. Tissues from tumors were mechanically cut up using razor blades and then dissociated at 37 degrees celsius for 2 h in RPMI 1640 medium supplemented with collagenase type IV (2

	mg/mL), DNase (0.1 mg/mL), hyaluronidase (0.1 mg/mL), and BSA (2 mg/mL). To acquire single cells, cell suspensions were passed through 100-µm filters. For surface protein staining, cells were incubated in the dark with antibodies for 20 min at room temperature. For intracellular protein staining, cells were fixed with Fix/Perm solution (BD Biosciences), then washed with Perm/Wash buffer (BD Biosciences), and stained intracellularly for 20 min in the dark at room temperature.
Instrument	FACScalibur (Beckman Coulter CytoFLEX LX, Beckman)
Software	FlowJo V10.4
Cell population abundance	The purity of FACS-sorted samples was assessed by post-sort flow cytometry with a purity of sorted cells of > 90%.
Gating strategy	FSC and SSC gates were used to select for single cells. A live/dead cell gate based on Zombie Aqua™ Fixable Viability Kit (Biolegend) or Zombie UV™ Fixable Viability Kit (Biolegend) was used to exclude dead cells. For immunophenotyping of tumor tissues, CD45+ CD11b+ F4/80+ gate strategy was used to identify macrophages, CD45+ CD11b+ Gr-1+ gate strategy was used to identify myeloid-derived suppressor cells, CD45+ CD11c+ gate strategy was used to identify dendritic cells, CD45+ CD19+ gate strategy was used to identify B cells, and CD45+ NK1.1+ gate strategy was used to identify nature killer cells. For analysis of CD47 expression on tumor cells harvested from mice, as tumor cells were pre-labled with GFP, GFP+ cells were gated to identify tumor cells. For phagocytosis assay in vitro, co-cultured cells were stained with PE-conjugated F4/80 to identify macrophages. As tumor cells were labled with GFP, F4/80+ GFP+ events were gated to represente macrophages that had phagocytosed tumor cells.

☒ Tick this box to confirm that a figure exemplifying the gating strategy is provided in the Supplementary Information.
